# Vascularized and Perfusable Human Heart‐on‐a‐Chip Model Recapitulates Aspects of Myocardial Ischemia and Enables Analysis of Nanomedicine Delivery

**DOI:** 10.1002/adma.202418909

**Published:** 2025-07-26

**Authors:** Junyoung Kim, Xuening Zhang, Richard Wang, Adrian Najer, Qiao You Lau, Ana Cammack‐Najera, Jang Ah Kim, Yoo Kyung Kang, Ruoxiao Xie, Hyemin Kim, Kai Xie, Hyeonji Lim, Tae‐Eun Park, Jinmyoung Joo, Molly M. Stevens

**Affiliations:** ^1^ Department of Materials, Department of Bioengineering and Institute of Biomedical Engineering Imperial College London Prince Consort Road London SW7 2AZ UK; ^2^ Department of Physiology, Anatomy and Genetics Department of Engineering Science Kavli Institute for Nanoscience Discovery University of Oxford South Parks Road Oxford OX1 3QU UK; ^3^ Department of Biomedical Engineering Ulsan National Institute of Science and Technology (UNIST) Ulsan 44919 Republic of Korea; ^4^ Graduate School of Health Science and Technology Ulsan National Institute of Science and Technology (UNIST) Ulsan 44919 Republic of Korea; ^5^ Center for Genomic Integrity Institute for Basic Science Ulsan 44919 Republic of Korea

**Keywords:** heart‐remodeling, myocardial ischemia, nanomedicines, vascularized heart‐on‐a‐chip

## Abstract

Cardiovascular diseases (CVDs) are the leading cause of death worldwide. However, the pathophysiological mechanisms of CVDs are not yet fully understood, and animal models do not accurately replicate human heart function. Heart‐on‐a‐chip technologies with increasing complexity are being developed to mimic aspects of native human cardiac physiology for mechanistic studies and as screening platforms for drugs and nanomedicines. Here, a 3D human myocardial ischemia‐on‐a‐chip platform incorporating perfusable vasculature in direct contact with myocardial regions is designed. Infusing a vasoconstrictor cocktail, including angiotensin II and phenylephrine, into this heart‐on‐a‐chip model leads to increased arrhythmias in cardiomyocyte pacing, fibroblast activation, and damage to blood vessels, all of which are hallmarks of ischemic heart injury. To verify the potential of this platform for drug and nanocarrier screening, a proof‐of‐concept study is conducted with cardiac homing peptide‐conjugated liposomes containing Alamandine. This nanomedicine formulation enhances targeting to the ischemia model, alleviates myocardial ischemia‐related characteristics, and improves cardiomyocyte beating. This confirms that the vascularized chip model of human myocardial ischemia provides both functional and mechanistic insights into myocardial tissue pathophysiology and can contribute to the development of cardiac remodeling medicines.

## Graphical abstract

This novel 3D human ischemic heart‐on‐a‐chip, with perfusable vasculature directly interfaced with myocardial tissue, replicates key aspects of ischemic heart injury. It also demonstrates the targeted delivery and therapeutic efficacy of cardiac homing peptide‐conjugated liposomes, validating the platform for drug and nanomedicine screening in myocardial ischemia.

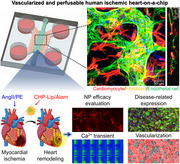

## 1 Introduction

According to the World Health Organization, cardiovascular diseases (CVDs) are a severe condition that causes 17.9 million deaths yearly worldwide, accounting for 32% of all deaths.^[^ ^1^ ^]^ Myocardial ischemia, a major pathological feature of CVDs, occurs when the coronary arteries are partially or completely blocked, leading to insufficient blood supply and oxygen deficiency. This deficiency results in a failure to meet the energy demands of heart tissue, causing cell death. The loss of cardiomyocytes (CMs) and tissue damage results in impaired contractility, as well as significant changes in electrophysiological health and metabolism. Persistent damage and malfunction can eventually lead to myocardial infarction, with the risk of heart failure.^[^ ^2^ ^]^ Despite the severity of this disease, therapeutic prevention of eventual heart failure has long been limited to a few therapies, such as angiotensin‐convertingenzyme inhibitors, beta‐blockers, angiotensin receptor blockers, and diuretics. Most therapeutic research efforts focus on temporarily mitigating symptoms and enhancing heart function, but do not actively reduce the risk of heart failure. This clearly highlights the necessity to develop novel therapeutics that offer long‐term effectiveness in improving cardiac function to prevent heart failure.^[^ ^3^ ^]^ Nanomedicines that transport heart‐remodeling drugs to diseased tissues have clinical therapeutic potential for certain CVDs. However, identifying the appropriate combination of drug, nanocarrier, and targeting functionality for CVDs is a complex challenge, and research is significantly lagging behind the advances of nanotherapeutics in other disease areas such as cancer.^[^ ^4^ ^]^

The development and testing of alternative treatments for CVDs require suitable preclinical disease models. Animal models present challenges due to inter‐species differences in heart rate, electrocardiogram duration, calcium (Ca^2+^) homeostasis regulation, metabolism, and pharmacokinetics, as well as ethical concerns.^[^ ^5^ ^]^ Similarly, alternative 2D human in vitro models fail to replicate the complex physiology of human myocardial tissue.^[^ ^6^ ^]^ Recent advances in bio‐ and tissue engineering have now led to the development of organ‐on‐a‐chip technologies, which are used to better recapitulate and predict pathological mechanisms and responses of various human organs.^[^ ^7^ ^]^ In parallel, stem cell technology has enabled the differentiation of human embryonic stem cells and induced pluripotent stem cells (hiPSCs) into CMs. These differentiated CMs possess the ion channels, signaling, and contractile proteins necessary for excitation‐contraction coupling, closely mimicking human cardiac physiology.^[^ ^8^ ^]^ Despite their immature phenotypes in morphological and biochemical properties, several engineering approaches, such as mechanical^[^ ^9^ ^]^ and electrical^[^ ^10^ ^]^ stimulation, coculture with nonmyocytes,^[^ ^6^ ^]^ extracellular matrix (ECM),^[^ ^11^ ^]^ and dynamic flow,^[^ ^12^ ^]^ have been used to promote the maturation of human CMs and engineer more relevant myocardial tissue. These, in turn, have facilitated the application of differentiated CMs to generate 3D human‐engineered myocardial tissue, enabling studies of cardiac function, drug efficacy, and toxicity under various conditions that mimic pathological scenarios.^[^ ^13^ ^]^

Initial 3D models of healthy or diseased myocardial tissue were limited to culturing CMs alone^[^ ^14^ ^]^ or in coculture with cardiac fibroblasts (CFs),^[^ ^15^ ^]^ failing to recapitulate the full complexity of myocardial tissue. Recent studies have introduced vascular networks into human heart tissue models, but these are either non‐perfusable^[^ ^16^ ^]^ or, if perfusable, only replicate healthy cardiac physiology.^[^ ^17^ ^]^ Moreover, current vascularized and perfusable heart disease models rely on physically separated compartments or membrane interfaces between vascular and myocardial tissues,^[^ ^18^ ^]^ making it challenging to control the small vascular diameter and understand the pathophysiology of CVDs, particularly vascular remodeling. In contrast, our platform enables the spontaneous formation of perfusable vasculature embedded within a 3D myocardial tissue matrix, allowing for direct physical and biochemical interactions between vasculature and cardiomyocytes. This design facilitates a comprehensive assessment of both cardiac and vascular responses under ischemic conditions, including contractile function, vascular permeability, and fibrosis, representing a more pathophysiologically relevant in vitro model for studying CVD progression. Furthermore, this platform uniquely enables real‐time introduction of nanomedicines within a disease‐mimicking microenvironment, providing functional insights into both targeting efficiency and therapeutic efficacy.

**Figure 1:**
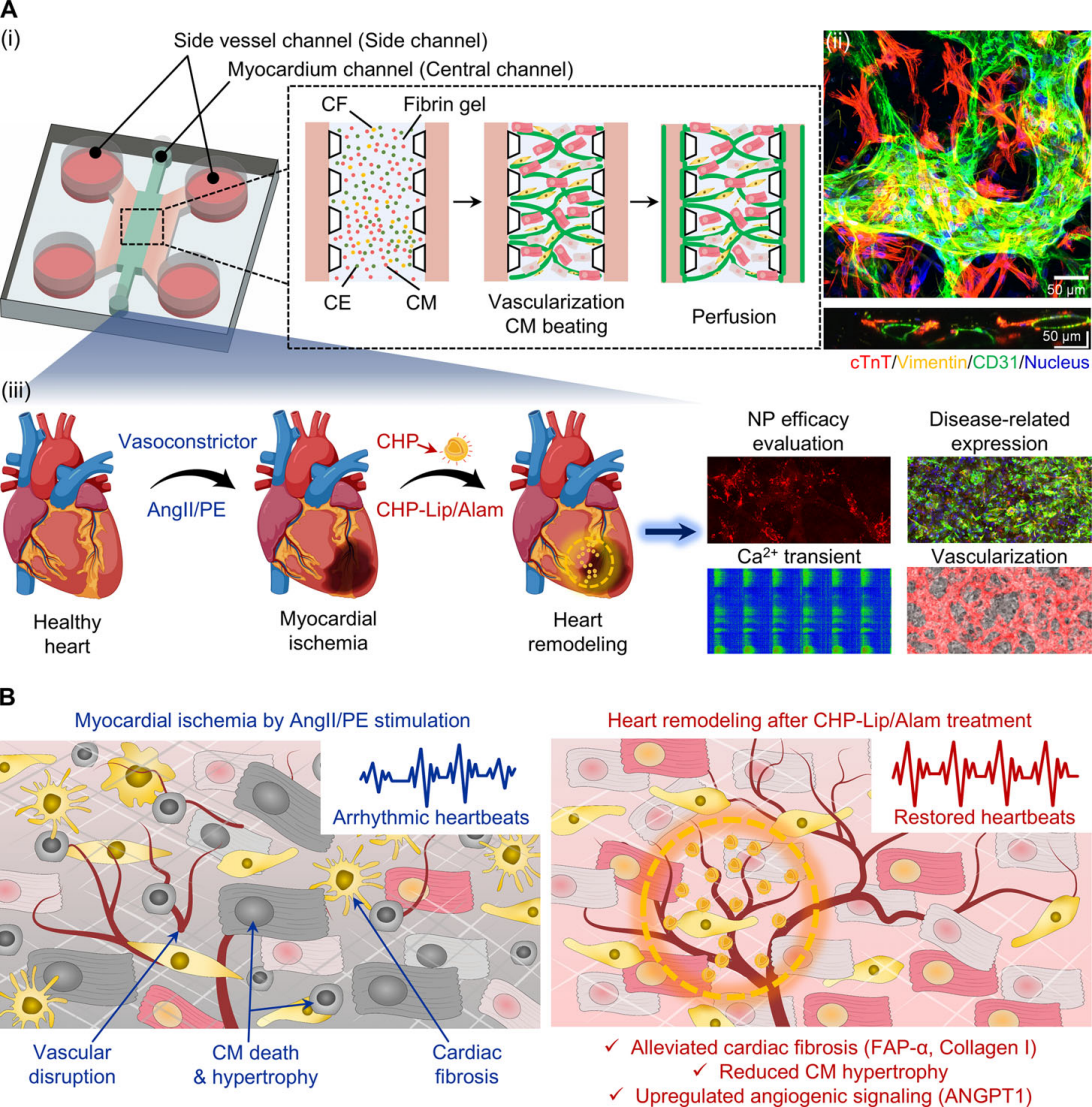
Development of a vascularized and perfusable human myocardial ischemia‐on‐a‐chip model. A‐i) Schematic illustration of the fabrication process using coculture of three cell types in a fibrin gel inside a microfluidic chip (hiPSC‐derived cardiomyocytes, CMs; cardiac fibroblasts, CFs; cardiac microvascular endothelial cells, CEs). A‐ii) Max‐intensity projection of a representative z‐stacked immunofluorescence confocal image showing an area in the central channel of the vascularized heart‐on‐a‐chip model (cTnT: red, vimentin: yellow, CD31: green, nuclei: blue; scale bar: 50 µm). A‐iii) Schematics of the heart to visualize the pathophysiology (AngII/PE infusion to mimic myocardial ischemia) and treatment (CHP‐modified and Alamandine‐loaded liposomes, CHP‐Lip/Alam) that was mimicked herein using our heart‐on‐a‐chip model. The analysis involved measuring the nanoparticle targeting efficacy and the response of disease‐related expression markers, Ca^2+^ transient profiles, and vascular morphology upon inducing myocardial ischemia and after treatment with nanomedicines. B) Detailed schematic that describes myocardial ischemia modeling and therapeutic recovery within the vascularized and perfusable heart‐on‐a‐chip platform. The left panel represents the ischemic state induced by AngII/PE infusion, characterized by arrhythmic CM beating, fibrosis, and vascular disruption. The right panel depicts the post‐treatment of CHP‐Lip/Alam, showing restored CM beating, reduced CM hypertrophy and fibrosis, and upregulation of angiogenesis‐related markers. Schematics created using Biorender (A‐iii).

Herein, we present a vascularized and perfusable heart‐on‐a‐chip incorporating myocardial tissue component cells within a 3D hydrogel using a microfluidic platform (**Figure** **1**A). Chronic exposure of the heart‐on‐a‐chip to a combination of vasoconstrictors, such as angiotensin II and phenylephrine (AngII/PE), demonstrated that our model recapitulated aspects of the pathophysiology of myocardial ischemia, including hypertension, exhibiting increased arrhythmic CM beating, CF activation, and vascular disruption (Figure 1B). As a proof‐of‐concept, we evaluated the potential of this model to analyze the effects of nanomedicine on disease progression in the chip. We demonstrated that cardiac homing peptide (CHP)‐conjugated liposomes containing Alamandine (CHP‐Lip/Alam) could alleviate myocardial ischemia‐related characteristics and improve CM beating function in our model (Figure S1, Supporting Information). Thus, our vascularized and perfusable human ischemic heart‐on‐a‐chip model provides valuable insights into the mechanisms of cardiac disease progression and has significant potential for accelerating the development and pre‐screening of effective cardiac remodeling therapies.

## 2 Results and Discussion

### 2.1 Recapitulation of Aspects of Myocardial Ischemia in Vascularized and Perfusable 3D Human Heart‐on‐a‐Chip

Native cardiac tissue is mainly composed of CMs that provide contractility (20∼40%), and nonmyocytes, including CFs and cardiac endothelial cells (CEs), which consist of over 50% of the cells in the myocardial tissue,^[^ ^19^ ^]^ play essential roles in cardiac development, homeostasis, and pathogenesis.^[^ ^20^ ^]^ To mimic physiologically relevant conditions, we established a 3D vascularized and perfusable human heart‐on‐a‐chip by encapsulating hiPSC‐derived CMs, primary cardiac human microvascular CEs, and primary human CFs at a 6:5:1 ratio^[^ ^16^, ^17^ ^]^ in a fibrin gel within a microfluidic platform.^[^ ^21^ ^]^ hiPSC‐derived CMs were successfully differentiated using Wnt signaling modulation protocols,^[^ ^8^ ^]^ with over 90% of cells expressing cardiac troponin T (cTnT) (Figure S2, Supporting Information). Fibrin was selected as the hydrogel platform based on its wide use in studying the contractility of CMs^[^ ^22^ ^]^ and its favorable properties that facilitate vascularization in 3D tissue.^[^ ^21^, ^23^ ^]^ After 4 days of coculturing these cellular components in the central channel of the microfluidic platform (myocardial channel in Figure 1A‐i), CMs exhibited spontaneous beating, and well‐organized vascularization by CEs surrounded by CMs and CFs was observed. CEs were then introduced through the side vessel channels to form perfusable anastomoses with the vessels in the central channel, thereby creating simple inlets to the complex vascular structure (Figure 1A‐i). These CD31‐positive vessels exhibited lumen structure in the engineered myocardial tissue composed of cTnT‐positive CMs and vimentin‐positive CFs, as verified by 3D z‐stacked confocal imaging (Figure 1A‐ii; Figure S3, Supporting Information), with a tissue thickness ranging from 120 to 150 µm confirmed by 3D reconstruction. We also confirmed that our vascularized heart‐on‐a‐chip demonstrated chemical interactions between cellular components as well as direct physical contact during beating (Video 1, Supporting Information). Furthermore, the platform is readily perfusable, as demonstrated by the introduction of fluorescent markers, which were observed to flow unobstructed throughout the vascular network (Video 2, Supporting Information). To confirm the physiological relevance of this perfusion, we assessed tissue hypoxia levels under three different conditions: 1) CMs only, 2) a vascularized but non‐perfusable, and 3) vascularized and perfusable heart‐on‐a‐chip models. The vascularized and perfusable model exhibited significantly reduced levels of hypoxia compared to the other two groups, confirming the functional contribution of the perfused vasculature to oxygen delivery and myocardial tissue homeostasis (Figure S4, Supporting Information).

After establishing the 3D human heart‐on‐a‐chip model, conditions of myocardial ischemia were mimicked by treating with a vasoconstrictor cocktail composed of AngII and PE. AngII is a bioactive effector of the renin‐angiotensin system,^[^ ^24^ ^]^ and PE is an α1‐adrenergic receptor agonist,^[^ ^25^ ^]^ which acts as a potent vasoconstrictor while increasing afterload through the overexpressed hypertension signaling pathway in chronic exposure. This combination is commonly used to induce ischemia as the synergistic stress inducers of cardiac pathogenesis, such as cardiac hypertrophy and fibrosis, leading to predictable heart failure in experimental animal models.^[^ ^26^ ^]^ We investigated the impact of vasoconstrictors on cardiac contractility in relation to heart function, fibrosis, and vascular disruption associated with heart pathophysiology. Additionally, we evaluated the heart‐remodeling efficacy of CHP‐Lip/Alam by assessing the change in Ca^2+^ transient profiles and disease‐related markers after treatment with model nanomedicines (Figure 1Aiii,B).

### 2.2 Increased Arrhythmia in 3D‐Engineered Myocardial Tissue Treated with AngII/PE

**Figure 2:**
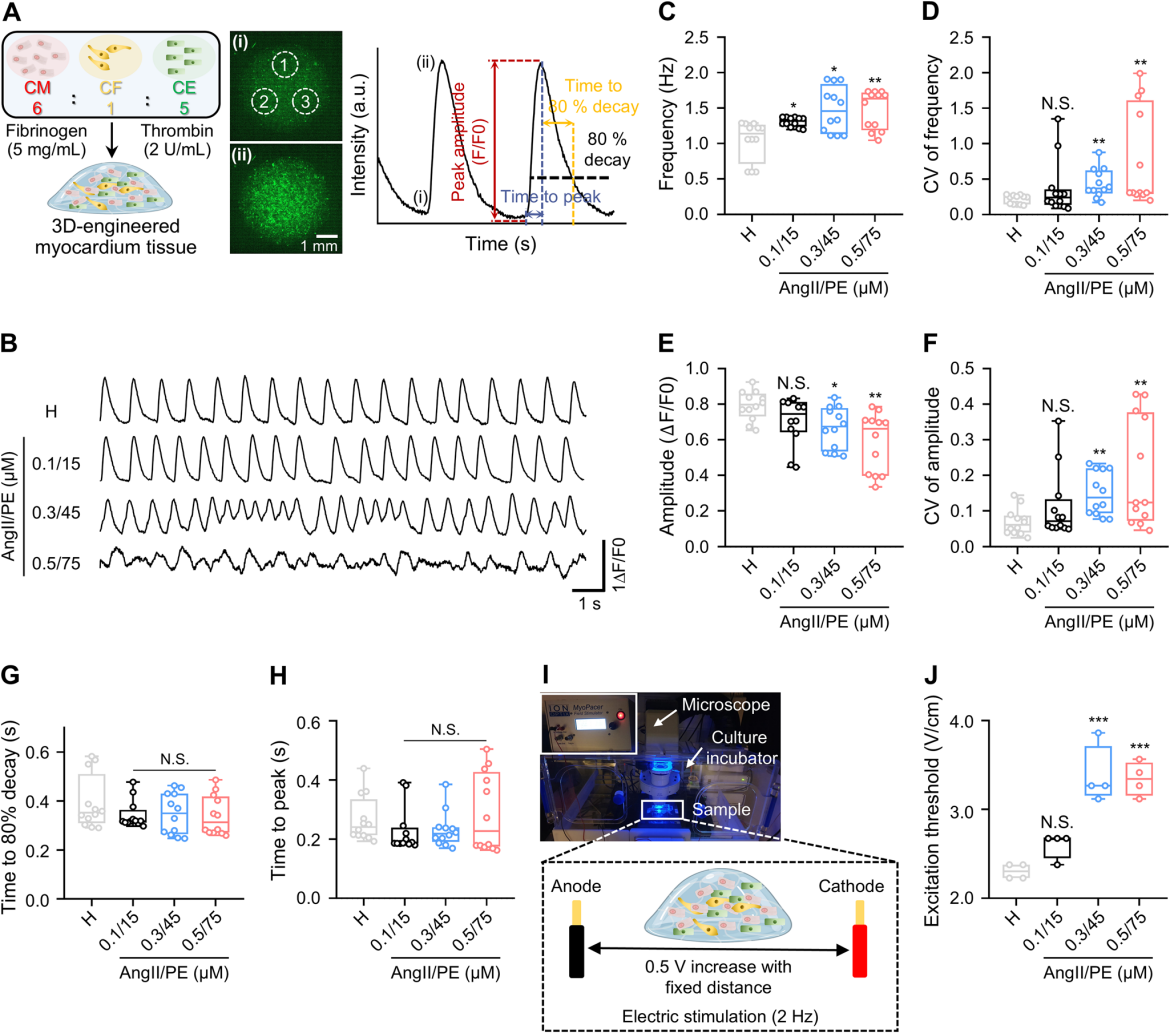
Vasoconstrictor infusion causes increased arrhythmia and poor electrical connectivity in our 3D‐engineered ischemic myocardial model. A) Schematic of 3D‐engineered myocardial tissue that allows measurements of Ca^2+^ transients of 3 ROIs per tissue, achieved by coculture of GCaMP hiPSC‐derived CMs, CFs, and CEs, with i and ii corresponding to baseline and peak intensity of green fluorescence signal. Scale bar, 1 mm. The graph illustrates the contraction parameters derived from the green fluorescence baseline and peak intensity. B) Representative Ca^2+^ indicator fluorescence traces of beating CMs within our 3D‐engineered myocardial tissues. C–H) Quantification of contraction parameters for our healthy (denoted as H) and diseased (vasoconstrictor infusion with AngII 0.1–0.5 µm and PE 15–75 µm) 3D‐engineered myocardial tissues: frequency (C); coefficient of variation (CV) of frequency (D); amplitude (E); CV of amplitude (F); time to 80% decay (G); and time to peak (H). Each point represents data from 1 region of interest (ROI) per tissue. Total *n* = 12 ROIs from 4 independent tissues. I) Image and schematic of the setting used for measuring the excitation threshold of 3D‐engineered myocardial tissue with 2 Hz frequency electrical stimulation and gradual voltage increase by 0.5 V increments, with a fixed distance between electrodes. J) The excitation threshold of 3D‐engineered myocardial tissues obtained from experiments as shown in (I). Each point represents data from one tissue (*n* = 4 biological independent samples). One‐way ANOVA with post hoc Dunnett's test (H, J) or Kruskal‐Wallis test with Dunn's test (C, D, E, F, G) was used to compare the difference of each AngII/PE‐treated sample with the healthy condition after normality test; N.S., not significant; *, *p* < 0.05; **, *p* < 0.01; ***, *p* < 0.001. The box plot represents the central line, denoting the median value, while the box contains the 25th to 75th percentiles of the dataset, with whiskers marking the maximum and minimum values. Schematics created using Biorender (A and I).

Cardiac dysfunction is associated with Ca^2+^‐handling abnormalities in CMs.^[^ ^27^ ^]^ To determine a baseline activity level and assess the effect of vasoconstrictors on Ca^2+^ handling, we assessed the spontaneous Ca^2+^ transients of CMs within the myocardial tissue of the heart‐on‐a‐chip before and after infusing various concentrations of AngII/PE. These tissues included CMs differentiated from GCaMP hiPSC, which are genetically modified to express a fluorescent Ca^2+^ indicator that allows real‐time visualization of Ca^2+^ dynamics.^[^ ^28^ ^]^ As reported in previous in vivo work,^[^ ^26^ ^]^ the concentrations of AngII/PE used for 7‐day treatments ranged from 0.1 to 0.5 µm for AngII and 15 to 75 µm for PE, which served as the baseline for our study. Based on the baseline and peak intensity from the green fluorescence signal measured for the GCaMP hiPSC‐derived CMs in the heart‐on‐a‐chip platform, contractility parameters such as frequency, amplitude, time to 80% decay, and time to peak were calculated to evaluate contractility consistency (**Figure** **2**A). Spontaneous contraction analysis revealed that AngII/PE disrupted intracellular Ca^2+^ homeostasis in a concentration‐dependent manner. (Figure 2B). AngII/PE induced a beating frequency increment from 1.04 to 1.48 Hz, lowering the amplitude by 0.74 times compared to the healthy condition (abbreviated as H) (Figure 2C,E). Furthermore, AngII/PE infusion resulted in an elevated coefficient of variation (CV, the ratio of the standard deviation to the mean) of both frequency and amplitude (Figure 2D–F). This resembled aspects of myocardial ischemia, characterized by irregular and arrhythmic contractile patterns due to Ca^2+^ mishandling. This observation was further consistent with in vivo studies showing hypertension induced by vasoconstrictors, which caused myocardial ischemia with irregular heartbeats.^[^ ^29^ ^]^ However, the time to 80% decay and the time to peak remained unchanged in our model (Figure 2G,H).

The excitation threshold was measured to assess the change in CM intercellular electrical connectivity. After evaluating the spontaneous contractility frequency, the electrical stimulation was set at 2 Hz, which is higher than the spontaneous CM contractility frequency, to ensure pacing capture without causing arrhythmia. The stimulation voltage was gradually increased in 0.5 V increments to determine the minimum stimulation voltage required to induce capture of externally paced contractions at a set pacing frequency (Figure 2I). Engineered myocardial tissue treated with AngII/PE showed 1.13, 1.47, and 1.45 times higher excitation thresholds than the healthy condition for the three different AngII/PE concentrations, indicating reduced overall tissue excitability upon treatment. Such increased excitation thresholds may be associated with altered CM coupling, increased heterocellular junctions, and/or higher ECM density^[^ ^30^ ^]^ (Figure 2J). Taken together, these results demonstrate the utility of the heart‐on‐a‐chip model in recapitulating several pathophysiologically relevant functional changes induced by vasoconstrictor infusion, including arrhythmia and significantly reduced intercellular electrical conductivity.

### 2.3 Chronic Exposure to AngII/PE Replicates Hallmarks of Cardiac Fibrosis in 3D‐Engineered Myocardial Tissue

**Figure 3:**
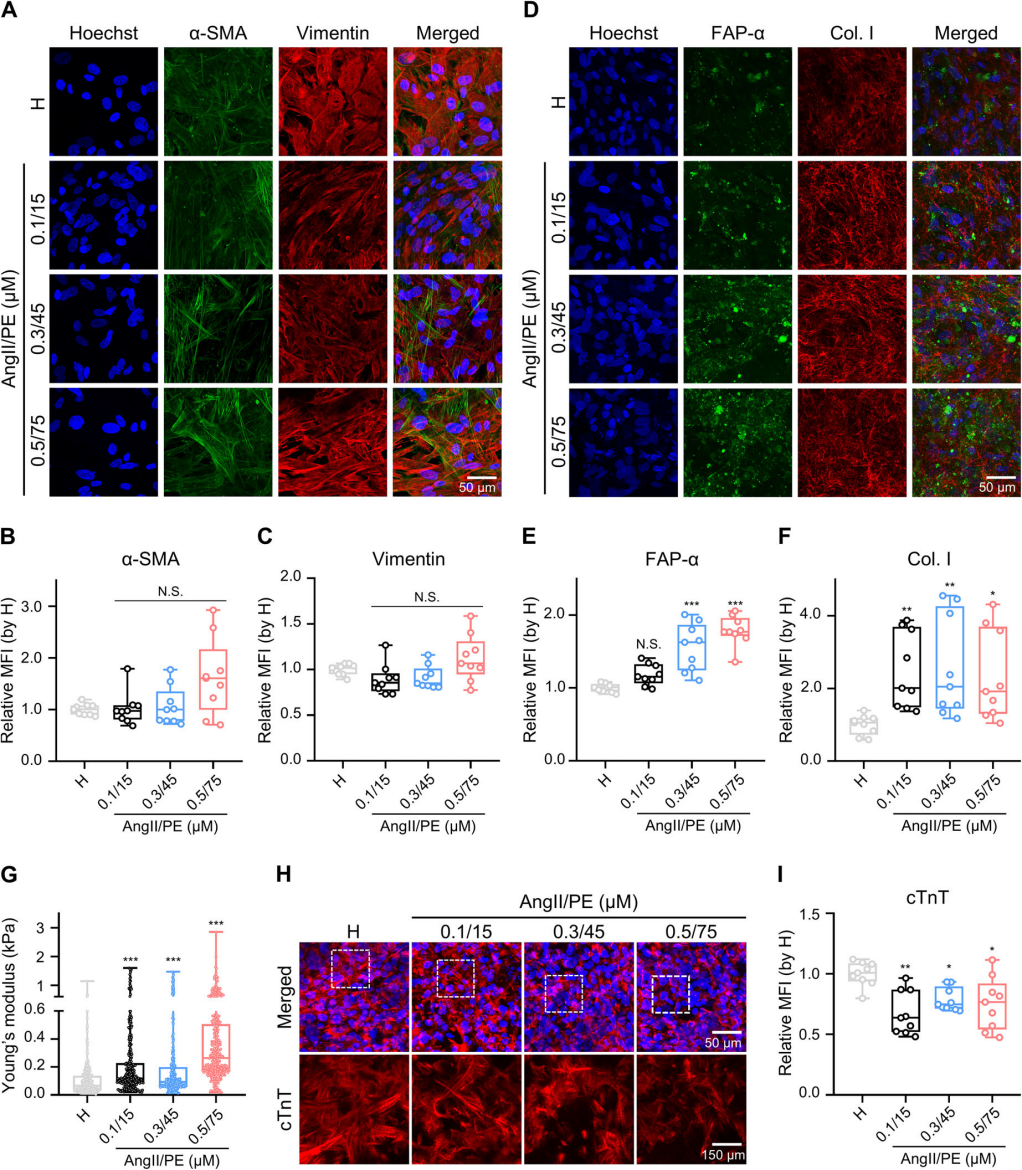
Markers of cardiac fibrosis, a hallmark of myocardial ischemia, are detected in 3D‐engineered myocardial tissues after the addition of vasoconstrictors. A–F) Representative z‐stacked immunofluorescence confocal images (max. intensity projections) and quantification of alpha‐smooth muscle actin (α‐SMA, green) and vimentin (red) (A‐C) and fibroblast activation protein‐α (FAP‐α, green) and type I collagen (Col. I, red) (D‐F) in 3D‐engineered myocardial tissue (Hoechst 33342: blue; Scale bar: 50 µm). Each point represents data from 1 ROI per tissue. Total *n* = 9 ROIs in 3 independent tissues. G) Average tissue stiffness of 3D‐engineered myocardial tissue measured by atomic force microscopy. Each point represents data from a single measurement of a randomly selected point in the tissue. Total *n* ∼ 300 measurements for 3 independent tissues. H) Representative z‐stacked immunofluorescence confocal images (max. intensity projections) of cardiac troponin T (cTnT, red) in 3D‐engineered myocardial tissue (nuclei: blue). Top: Merged image showing cTnT and nuclei (scale bar: 50 µm). Bottom: Magnified view of the white dotted region in the top panel, showing cTnT signal only (scale bar: 150 µm). I) Quantification of cTnT in 3D‐engineered myocardial tissue. Each point represents data from 1 ROI per tissue. Total *n* = 9 ROIs in 3 independent tissues. Experimental groups of 3D‐engineered myocardial tissues contained healthy (denoted as H, no treatment with vasoconstrictors) and diseased conditions, with the addition of different concentrations of vasoconstrictors (AngII 0.1–0.5 µm and PE 15–75 µm). One‐way ANOVA with post hoc Dunnett's test (B, F) or Kruskal‐Wallis test with Dunn's test (C, E, G, I) were used to compare the difference of each AngII/PE‐treated sample with the healthy condition after normality test; N.S., not significant; *, *p* < 0.05; **, *p* < 0.01; ***, *p* < 0.001. The box plot represents the central line, denoting the median value, while the box contains the 25th to 75th percentiles of the dataset, with whiskers marking the maximum and minimum values.

Cardiac fibrosis is one of the main features of myocardial tissue pathogenesis. Fibrosis in the heart is associated with myofibroblast differentiation from resident CFs within the native myocardium. This process leads to the excessive deposition of ECM, such as collagen, which substantially contributes to structural disorder and contractile dysfunction.^[^ ^31^ ^]^ Hence, we validated the effect of AngII/PE in our model to confirm the observation of fibrosis after 7 days of exposure (**Figure** **3**A–F, Supporting Information). Vimentin, a type III intermediate filament cytoskeletal protein, is a typical marker of mesenchymal lineages, including CFs and CEs, but not CMs,^[^ ^32^ ^]^ whilst α‐smooth muscle actin (α‐SMA) and fibroblast activation protein‐α (FAP‐α) are cardiac myofibroblast markers. α‐SMA is related to increased contractile activity of myofibroblasts, which can be used as a marker for fibrogenic activity.^[^ ^33^ ^]^ FAP‐α is a cell‐surface glycoprotein expressed by activated fibroblasts during wound repair in tissue fibrosis in tumors and in acute myocardial infarction.^[^ ^34^ ^]^ Despite treating our engineered myocardial tissues with different concentrations of AngII/PE, no significant changes in vimentin nor α‐SMA expression were found (Figure 3B,C). However, tissues treated with 0.3/45 and 0.5/75 µm AngII/PE displayed a 55.6% and 78.8% increase in FAP‐α intensity relative to the healthy condition, respectively (Figure 3E). Increased expression of FAP‐α indicated that myofibroblasts were becoming more abundant in the AngII/PE‐treated heart‐on‐a‐chip myocardial tissues, similar to native cardiac fibrotic tissue. Additionally, AngII/PE infusion also caused significantly increased type I collagen deposition in our engineered myocardial tissues, showing more than 2.33‐fold increases for all concentrations of AngII/PE versus the non‐treated healthy condition (Figure 3F).

Next, we assessed the average stiffness of living engineered myocardial tissue under different culture conditions using atomic force microscopy. The baseline stiffness of healthy engineered tissues was observed to be 106.4 Pa on average, whereas the diseased model possessed increased stiffness of 225.2, 199.1, and 372.3 Pa for AngII/PE concentrations of 0.1/15, 0.3/45, and 0.5/75 µm, respectively (Figure 3G). This might be related to the measured excessive deposition of type I collagen in AngII/PE‐treated tissues (Figure 3F). Moreover, engineered tissues treated with AngII/PE demonstrated a broader stiffness variation, ranging from 13.2 to 2851.6 Pa, compared to healthy conditions, which ranged from 5.2 to 1142.0 Pa, indicating more heterogeneous properties for the fibrosis model, a characteristic also typical of native fibrotic tissue.^[^ ^35^ ^]^ Hence, the increased stiffness and stiffness variation observed in AngII/PE‐treated tissues replicated important aspects of cardiac fibrosis. It should be noted that the measured stiffness range for our model is comparable to other engineered vascularized myocardial‐like tissues in fibrin‐based scaffolds,^[^ ^36^ ^]^ but deviates from the typical in vivo stiffness range (healthy: ≈18 kPa, cardiac fibrosis: ≈55 kPa).^[^ ^37^ ^]^ This deviation is likely a result of different cell densities, total ECM composition, and structural variation between the in vitro and in vivo tissue models.^[^ ^38^ ^]^ This suggests a need for future improvement in engineering cardiac tissue that better replicates the native myocardium in terms of stiffness.^[^ ^39^ ^]^

Lastly, we characterized the expression of cTnT, which plays a vital role in regulating cardiac muscle contraction, and where decreased cTnT expression has been previously associated with ischemia‐reperfusion injury or myocardial infarction in vivo, representing damage to the CMs.^[^ ^40^ ^]^ Our heart‐on‐a‐chip platform successfully demonstrated a significant decrease in intracellular cTnT expression when treated with AngII/PE compared to the healthy control (Figure 3H,I). Besides reduced expression of cTnT in damaged CMs, this might also be related to a reduction in CMs due to increased CM death (Figure S5, Supporting Information). Overall, these findings demonstrate that 3D‐engineered myocardial tissue, combined with AngII/PE infusion, recreates hallmark aspects of heart disease pathophysiology comparable to clinical observations.

### 2.4 Vascular Dysfunction in 3D Human Myocardial Ischemia‐on‐a‐Chip Model

Structural abnormalities in blood vessels due to plaque accumulation in the endothelium and other pathophysiological causes can impede proper blood flow, leading to the progression of CVDs such as atherosclerosis, myocardial ischemia, and myocardial infarction. Therefore, understanding changes in the vasculature of myocardial tissue during the progression of heart disease is crucial for comprehending the mechanisms of CVDs.^[^ ^41^ ^]^ Despite numerous in vitro systems recreating various heart diseases using CEs, only a few studies have extensively explored changes in vessel structure and functionality under conditions mimicking pathological conditions.^[^ ^17^, ^36^ ^]^ Our heart‐on‐a‐chip platform is well‐suited for studying vascular disruption due to environmental changes, as it is based on direct physical contact between the vasculature and the engineered myocardial tissue.

**Figure 4:**
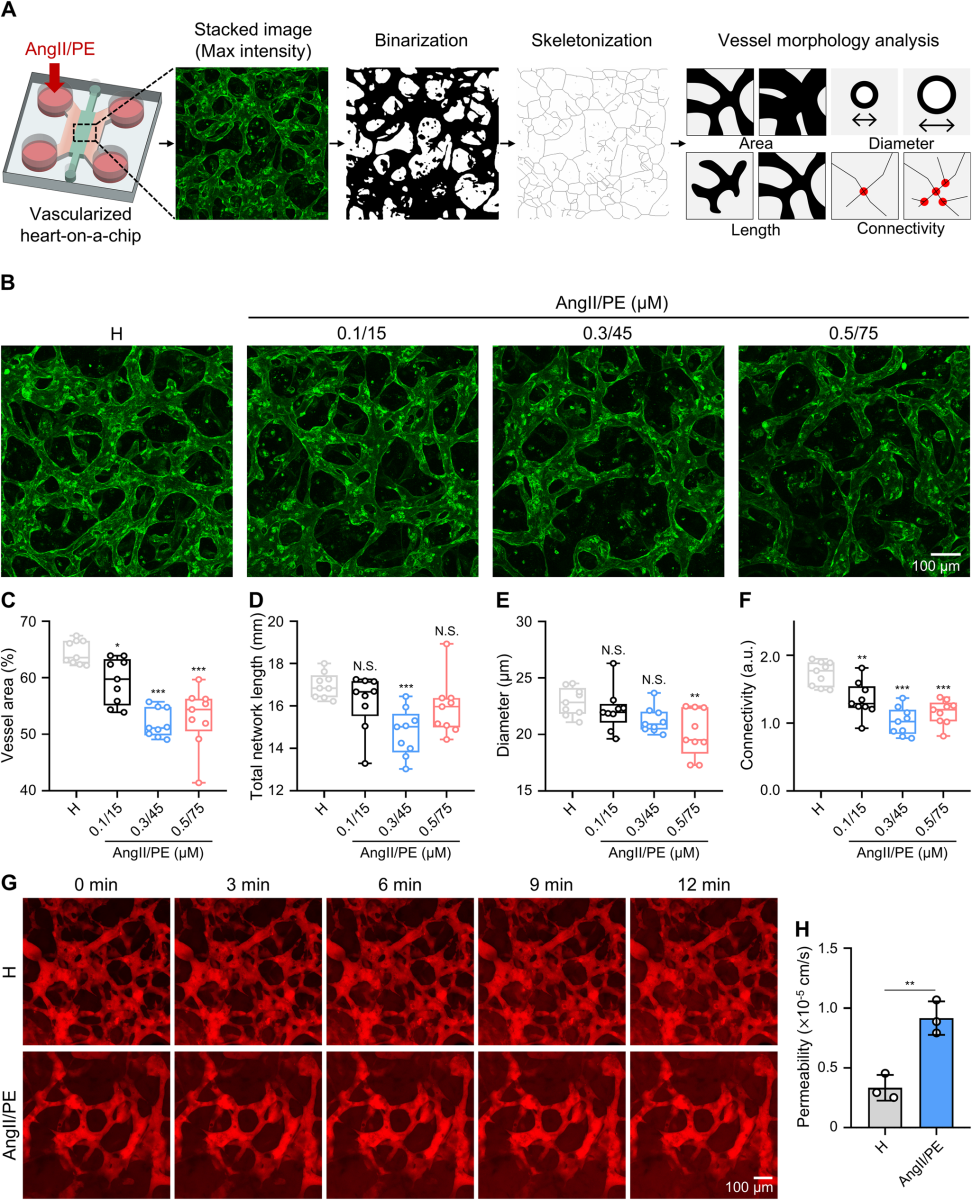
Vascular disruption is exhibited in 3D vascularized heart‐on‐a‐chip models after the infusion of vasoconstrictors. A) Schematics of protocol steps for measuring vessel morphology parameters from z‐stacked confocal images of 3D vessels in heart‐on‐a‐chip. B–E) Representative z‐stacked immunofluorescence confocal images of CD31‐stained vessels (max. intensity projections; Scale bar: 100 µm) and quantification of vessel morphology parameters from the images, comparing the healthy condition to platforms treated with different concentrations of vasoconstrictors (AngII 0.1–0.5 µm and PE 15–75 µm): diameter (C); vessel area (D); connectivity (E); total network length (F). Each point represents data from 1 ROI in one chip. Total *n* = 9 ROIs in 3 independent chips. G,H) Representative z‐stacked fluorescence confocal images (max. intensity projections) after infusion of fluorescent 70 kDa dextran (red) for 0, 3, 6, 9, and 12 min and associated permeability values for diffusion across the vessels as observed in selected ROIs in healthy 3D heart‐on‐a‐chip models and models treated with 0.3/45 µm AngII/PE (Scale bar: 100 µm). Each point represents data from one independent chip. One‐way ANOVA with post hoc Dunnett's test (C, D, E) or Kruskal‐Wallis test with Dunn's test (F) was used to compare the difference of each AngII/PE‐treated sample with the healthy condition after the normality test and the two‐tailed unpaired Student's t‐test was used to compare the means of two groups (H); N.S., not significant; *, *p* < 0.05; **, *p* < 0.01; ***, *p* < 0.001. The box plot (C–F) represents the central line denoting the median value, while the box contains the 25th to 75th percentiles of the dataset with whiskers marking the maximum and minimum values, and a bar graph (H) represents mean ± standard deviation.

We used our vascularized and perfusable heart‐on‐a‐chip model to investigate the effect of AngII/PE on vascular disruption in vitro. After treating the heart‐on‐a‐chip models with varying concentrations of AngII/PE for 7 days via infusion through the side channel inlets, the vessel morphology of the vasculature was evaluated via z‐stacked 3D vasculature immunofluorescence confocal imaging (**Figure** **4**A,B). Quantitative evaluation of these images confirmed that vessel morphology was successfully affected by AngII/PE infusion in our heart‐on‐a‐chip platform. Healthy conditions demonstrated a baseline average of 64.5% microvascular network (MVN) coverage area, whereas AngII/PE infusion with increasing concentrations significantly reduced the area to 59.4, 51.9, and 53.0%, respectively (Figure 4C). In addition, the total network length decreased upon AngII/PE infusion (Figure 4D), while the average vessel diameter also significantly decreased between the healthy condition (22.9 ± 1.2 µm vessel diameter), and the highest 0.5/75 µm AngII/PE concentration (20.1 ± 2.1 µm vessel diameter), potentially representing a vasoconstriction effect (Figure 4E). We also observed lower connectivity under disease conditions compared to the healthy condition (Figure 4F). These observations on our vascularized heart‐on‐a‐chip platform are consistent with clinical data showing lower MVN diameter and density in pathological heart disease conditions,^[^ ^42^ ^]^ highlighting the utility of our platform in disease modeling. Among the tested concentrations, AngII (0.3 µm) and PE (45 µm) were selected for subsequent experiments as they robustly induced key pathological features—such as abnormal Ca^2+^ handling, fibrosis, and vascular disruption—while higher concentrations did not lead to further pathological progression.

Leaky vasculature, as previously reported, is another hallmark indication of ischemic heart injury in vivo.^[^ ^43^ ^]^ Hence, we next examined the vascular permeability of our heart‐on‐a‐chip platform by infusing 70 kDa dextran, a tracking molecule. Comparing the healthy condition to the 0.3/45 µm AngII/PE‐treatment, our heart‐on‐a‐chip platform successfully showed that diseased vessels exhibited a permeability of (9.2 ± 1.4) × 10^−6^ cm s^−1^, 2.7 times higher than that of the healthy condition at (3.3 ± 1.1) × 10^−6^ cm s^−1^ (Figure 4G,H). In addition to permeability changes, we also observed increased vimentin expression in CD31‐positive endothelial cells under the 0.3/45 µm AngII/PE condition, suggesting a phenotypic transition consistent with endothelial‐to‐mesenchymal transition (Figure S6, Supporting Information). These results suggest that exposure to AngII/PE leads to dysfunction through vascular disruption and hyperpermeability. This increase in permeability can potentially be leveraged for nanomedicine delivery to ischemic heart tissue, similar to the enhanced permeation and retention effect utilized for the passive delivery of nanoparticles through the leaky vasculature of some solid tumors.^[^ ^44^ ^]^ Hence, we next sought to evaluate the ischemic heart‐on‐a‐chip platform to analyze a drug delivery strategy and quantify the effects of the accompanying drug action.

### 2.5 Cardiac Homing Peptide‐Conjugated Liposomes Containing Alamandine Reduce Characteristics of Myocardial Ischemia in 3D Human Heart‐on‐a‐Chip Model

**Figure 5:**
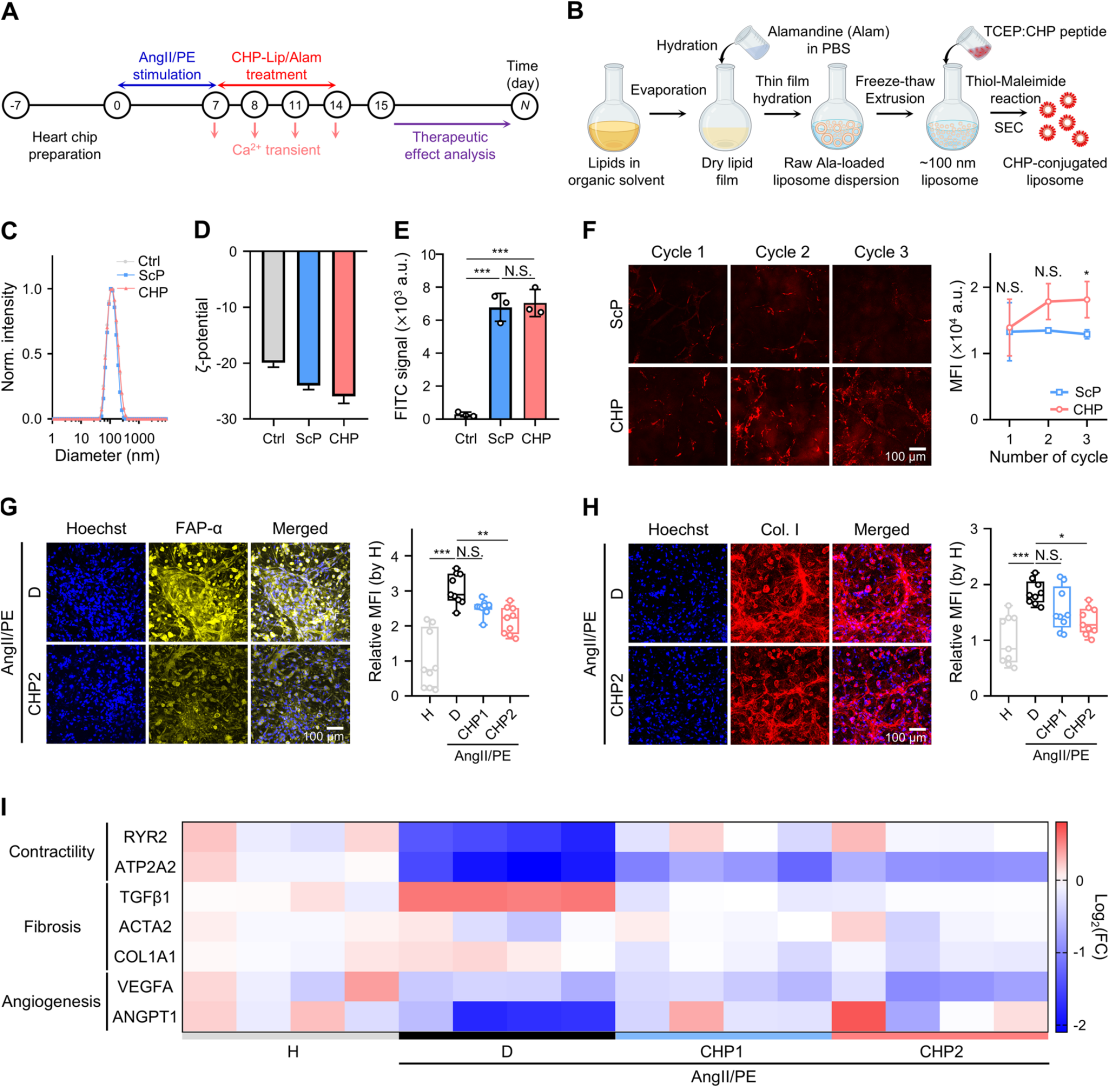
Administration of liposomal nanomedicine (CHP‐Lip/Alam) attenuates myocardial ischemia‐related characteristics in 3D human myocardial ischemia‐on‐a‐chip. A) Experimental timeline of nanomedicine testing using 3D vascularized heart‐on‐a‐chip and CHP‐Lip/Alam. B) Schematic of protocol steps to produce CHP‐Lip/Alam. C,D) Liposome characterization using DLS showing average hydrodynamic diameter by intensity (C) and zeta potential (D) (*n* = 3 technical replicates). E) Quantification of FITC intensity of liposomes conjugated with FITC‐labelled ScPs and CHPs. Each point represents data from each sample (*n* = 3 biological replicates). F,G) Representative z‐stacked immunofluorescence confocal images and quantification of fluorescence intensity of tissue‐accumulated liposomes (max. intensity projection, red) conjugated with ScPs and CHPs (300 µg mL^−1^) after 3 cycles of infusion in the 3D human myocardial ischemia‐on‐a‐chip model, which was induced by infusion of 0.3/45 µm AngII/PE (Scale bar: 100 µm, *n* = 3 independent chips).(H–K) Representative z‐stacked immunofluorescence confocal images (max. intensity projection) and image quantification of fibroblast activation protein‐α (FAP‐α, yellow, H‐I) and type I collagen (Col.I, red, J‐K) of 3D human myocardial ischemia‐on‐a‐chip models perfused with 0.3/45 µm AngII/PE and after treatment with different concentrations of CHP‐Lip/Alam. Hoechst 33342 (blue) indicates the nuclei. Each point represents data from 1 ROI in one chip. Total *n* = 9 ROIs in 3 independent chips. L) Heatmap of Log2 of the fold change in expression of contractility, fibrosis, and angiogenesis‐related genes obtained by reverse transcription quantitative PCR. Each sample was prepared by pooling *n* = 3 chips, which was repeated in *n* = 4 independent experiments. One‐way ANOVA with post hoc Tukey's test (E, I, K) or Sidak's test (G) were used to compare the difference among conditions after the normality test; N.S., not significant; *, *p* < 0.05; **, *p* < 0.01; ***, *p* < 0.001. The bar (D, E) and line graph (G) represent mean ± standard deviation, and box plot (I, K) represents the central line denoting the median value, while the box contains the 25th to 75th percentiles of the dataset with whiskers marking the maximum and minimum values. Schematics created using Biorender (B).

To verify whether our ischemic heart‐on‐a‐chip disease model can be utilized as a platform for drug and nanocarrier analysis, we performed a proof‐of‐concept experiment by infusing CHP‐Lip/Alam to demonstrate the targeting of these peptide‐functionalized liposomes to our engineered heart tissues, which resemble ischemic disease, versus healthy tissues. The drug cargo, Alamandine (Alam), is a heptapeptide (Ala‐Arg‐Val‐Tyr‐Ile‐His‐Pro) known to alleviate pressure overload and AngII‐induced hypertrophy by activating the AMP‐activated protein kinase–nitric oxide pathway via Mas‐related G protein‐coupled receptor member D in cellular components of myocardium.^[^ ^45^ ^]^ Alam can protect the heart from ischemia‐reperfusion injury by activating c‐Jun N‐terminal kinase and inhibiting nuclear factor kappa B.^[^ ^46^ ^]^ Herein, the treatment and analysis schedule first included heart‐on‐a‐chip preparation and subsequent administration of 0.3/45 µm AngII/PE overlapping with CHP‐Lip/Alam treatment daily for 7 days. CM contractility was measured throughout the liposome treatment procedure, and the drug‐induced remodeling efficacy in terms of protein and RNA expressions was analyzed at the end (**Figure** **5**A). Alam‐loaded liposomes were prepared using a thin film hydration method followed by freeze‐thaw cycles and extrusion (loading efficiency: 4.4 ± 1.0% as measured by UV‐vis spectrophotometry). The liposomes consisted of a mixture of 1,2‐distearoyl‐sn‐glycero‐3‐phosphocholine (DSPC), cholesterol, and 1,2‐distearoyl‐sn‐glycero‐3‐phosphoethanolamine‐N‐[maleimide(polyethylene glycol)‐5000] (DSPE‐PEG‐maleimide). We selected CHP (CSTSMLKAC) for conjugation to the liposome surface due to the previously found targeting ability of this CHP to ischemic myocardium via selective binding to CMs in ischemic tissue.^[^ ^47^ ^]^ A scrambled peptide (ScP, CSKTALSMC) was used as a negative and non‐targeting control. These peptides were conjugated to the liposomes through a thiol‐maleimide reaction between the cysteine at the peptide's termini and the maleimide groups on the liposomes (Figure 5B). After subsequent purification with size exclusion chromatography, dynamic light scattering (DLS) measurements confirmed that our liposomes were monodisperse with similar diameters of ≈ 110 nm regardless of peptide conjugation. The measurements indicated that peptide‐conjugated liposomes exhibited a slightly more negative zeta potential (−24.0 ± 0.7 mV and −25.9 ± 1.3 mV for ScP and CHP, respectively) compared to bare liposomes (−19.9 ± 0.8 mV) (Figure 5C,D). After purification, we further confirmed the successful peptide conjugation by comparing the fluorescence intensity between fluorescein isothiocyanate (FITC)‐labeled peptide‐conjugated and unconjugated liposomes (Figure 5E).

Following confirmation of successful peptide conjugation to our liposomes, we prepared and introduced DiD‐stained peptide‐conjugated liposomes (300 µg mL^−1^) into the ischemic heart‐on‐a‐chip platform through hydrostatically driven flow (3 cycles). We first compared the accumulation levels of ScP‐ and CHP‐conjugated liposomes after 30 min (3 cycles). Over time, CHP‐conjugated liposomes showed increased accumulation within the ischemic heart‐on‐a‐chip model, while ScP‐conjugated liposomes did not show such changes (Figure 5F,G). In healthy heart‐on‐a‐chip models under the same administration conditions, both ScP‐ and CHP‐conjugated liposomes showed comparably low accumulation (Figure S7, Supporting Information), which could be related to the lower vascular permeability (Figure 4H). To further support this observation, colocalization analysis with CD31‐positive vessels showed that a greater proportion of CHP‐conjugated liposomes were located outside the vasculature in the ischemic model (61.8 ± 5.3%) compared to the healthy model (45.3 ± 9.6%). The enhanced localization of CHP‐conjugated liposomes is likely attributable to the combination of both vascular remodeling in the diseased myocardial tissue and the targeting ability of CHP, which together promote selective liposome accumulation in the pathological regions (Figure S8, Supporting Information). These results align with several in vivo studies, indicating that CHP‐conjugated nanoparticles exhibit high targeting ability for ischemic myocardium,^[^ ^47^ ^]^ suggesting that our ischemic heart‐on‐a‐chip platform has the potential to evaluate the targeting abilities of new nanoparticles, functional moieties, and chemical reagents relevant to clinical heart disease modeling.

To assess the therapeutic remodeling potential of CHP‐Lip/Alam in our ischemic heart‐on‐a‐chip platform, we administered different concentrations of CHP‐Lip/Alam with 0, 250, and 500 µg mL^−1^ (total lipid concentration) denoted as D (Diseased), CHP1, and CHP2 in AngII/PE groups, respectively (Alam concentration: 0, 10.5, and 21.0 µg mL^−1^, respectively). Liposomes were perfused for 3 h through hydrostatically driven flow, followed by removal of non‐bound liposomes via washing with media and incubation for another 24 h. This procedure was repeated daily over 7 days. The Alamandine concentrations were selected based on various in vitro^[^ ^48^ ^]^ and in vivo^[^ ^48^, ^49^ ^]^ studies and benchmarked against CHP‐Lip/Alam cytotoxicity assay results (Figure S9, Supporting Information). After 7 days of treatment with two different concentrations of CHP‐Lip/Alam, we observed that the intensity of FAP‐α was reduced to 82.7% and 70.6% (Figure 5H,I), and type I collagen expression was reduced to 83.4% and 72.4% (Figure 5J,K), respectively, compared to the untreated ischemic condition, indicating significant alleviation of cardiac fibrosis markers in our model using CHP‐Lip/Alam treatment.

**Figure 6:**
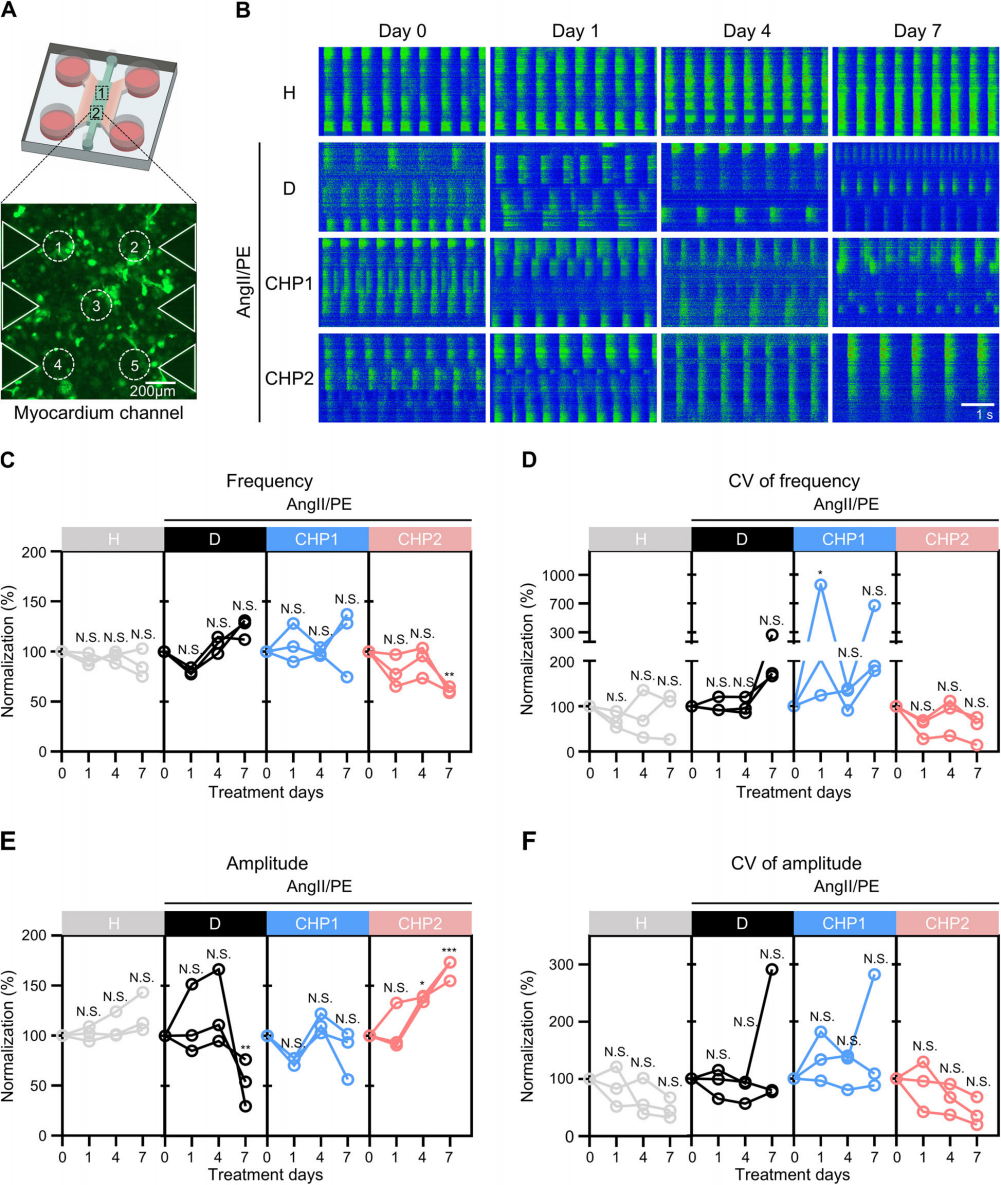
Administration of liposomal nanomedicine (CHP‐Lip/Alam) alleviates arrhythmic CM beating in a 3D human myocardial ischemia‐on‐a‐chip model. A) Schematic representation of regions of interest (ROIs) selected for Ca^2+^ transient analysis in the myocardium channel of the heart‐on‐a‐chip. 2 regions were defined per chip (region 1 and 2), and 5 ROIs were selected within each region, resulting in 10 ROIs per chip for quantitative analysis. B) Representative tracing of 2D Ca^2+^ transients in the healthy conditions without CHP‐Lip/Alam treatment (“H” panel) and the myocardial ischemia conditions after treatment with different concentrations of CHP‐Lip/Alam (“D, CHP1, and CHP2” panels in AngII/PE group) in the 3D heart‐on‐a‐chip. C–F) Profiles of extracted Ca^2+^ transient parameters (amplitude, CV of amplitude, frequency, and CV of frequency) in the 3D heart‐on‐a‐chip under different conditions for the next 7 days. Each point represents data from each chip. Total *n* = 30 ROIs in 3 independent chips. All the data were normalized to the data from each chip before CHP‐Lip/Alam treatment on day 0. Two‐way ANOVA with post hoc Dunnett's test (B, C, D, E) was used to compare each condition to its respective baseline (Day 0) after the normality test; N.S., not significant; *, *p* < 0.05; ***, *p* < 0.001.

Next, we analyzed the expression of contractility, fibrosis, and angiogenesis‐related mRNAs for various conditions of the heart‐on‐a‐chip model (Figure 5L; Figure S10, Supporting Information), which was obtained through gel lysis and cell collection (Figure S11, Supporting Information). Contractility‐related genes such as RYR2 and ATP2A2 exhibited significantly decreased expression in the ischemic heart‐on‐a‐chip model without CHP‐Lip/Alam treatment. This finding aligned well with the increased arrhythmic contractions due to Ca^2+^ handling abnormalities (Figure 2). Treatment with CHP‐Lip/Alam at both concentrations restored some of the expression of RYR2 and ATP2A2, while CHP2 performed slightly better than CHP1 (Figure S10A, Supporting Information). This suggests that CHP‐Lip/Alam has the potential to restore CM contractile function after AngII/PE‐induced ischemia (**Figure** **6**), providing additional utility for disease modeling. Among fibrosis‐related genes, only TGFβ1 showed significantly higher expression in the ischemic heart‐on‐a‐chip compared to healthy conditions, contrasting expression levels of ACTA2 and COL1A1. Upon treatment with CHP‐Lip/Alam at both concentrations, we observed a reduction in the expression of TGFβ1 to healthy levels, and a slight reduction in the expression of COL1A1, indicating that CHP‐Lip/Alam treatment suppressed AngII/PE‐induced cardiac fibrosis markers in this system (Figure S10B, Supporting Information). Likewise, the expression levels of the prominent angiogenesis‐related genes, such as VEGFA and ANGPT1, were significantly downregulated in the ischemic heart‐on‐a‐chip, whilst CHP‐Lip/Alam treatment restored ANGPT1 expression to healthy levels, although there were no significant changes observed in VEGFA (Figure S10C, Supporting Information). Altogether, these results suggested that CHP‐Lip/Alam treatment can alleviate pathological factors in our ischemic heart‐on‐a‐chip platform in a multifaceted way, thereby providing utility as a disease model in regenerative therapeutics.

Additionally, we measured the levels of secreted brain natriuretic peptide (BNP) in medium extracted from the heart‐on‐a‐chip platform for the various conditions at different time points. BNP secretion is considered one of the most reliable prognostic indicators of clinical heart failure and is a diagnostic marker for detecting cardiac events.^[^ ^50^ ^]^ In the disease condition group, the concentration of secreted BNP was significantly higher (≈4.25 times) compared to the healthy control group on day 0. As hypothesized, treating with CHP‐Lip/Alam at both concentrations significantly reduced BNP secretion (Figure S12, Supporting Information), consistent with our results for cardiovascular‐related proteins and mRNA expression (Figure 5H–L). Under high‐dose CHP‐Lip/Alam treatment (CHP2), the decrease in secreted BNP concentration was maintained over 7 days, eventually resulting in only slightly higher levels (1.38‐fold) compared to the healthy condition. AngII/PE infusion also activates the Renin‐angiotensin‐aldosterone system, which induces cardiac hypertrophy accompanied by cardiac fibrosis and heart failure in vivo.^[^ ^51^ ^]^ Interestingly, the cTnT‐positive CMs of the healthy heart‐on‐a‐chip showed a diameter of 31.3 ± 1.8 µm, while the CMs of the ischemic heart‐on‐a‐chip exhibited a significantly larger size of 34.2 ± 0.1 µm. When a high concentration of CHP‐Lip/Alam was applied to the ischemic heart‐on‐a‐chip model for 7 days, the size of CMs was reduced to 32.8 ± 0.8 µm, confirming that CHP‐Lip/Alam treatment under specific conditions can also mitigate cardiac hypertrophy (Figure S13, Supporting Information). This aligns with in vivo data that illustrated the role of AngII/PE and the therapeutic potential of Alam in cardiac hypertrophy.^[^ ^52^ ^]^ Hence, our ischemic heart‐on‐a‐chip platform accurately recreated aspects of the pathophysiological in vivo environment, and we demonstrated the potential of using this model for drug and nanocarrier analysis and screening for therapeutic heart remodeling applications.

### 2.6 Improvement of CM Beating Function after Treatment of 3D Human Myocardial Ischemia‐on‐a‐Chip Model with Liposomal Nanomedicine

After confirming that successful drug delivery via targeted liposomes ameliorates pathological markers related to ischemic myocardium, we further assessed whether CHP‐Lip/Alam could recover CM contractility to resolve arrhythmia induced by AngII/PE infusion (Figure 2). We analyzed changes in Ca^2+^ transients of CMs within our ischemic heart‐on‐a‐chip model, which reflect cardiac remodeling effects, as shown in Figure 5. First, in the ischemic condition without CHP‐Lip/Alam treatment, but with an additional 7 days of AngII/PE infusion, we observed a persistent increase in frequency and a tendency for CV of frequency and amplitude to either maintain or increase, leading to worsening arrhythmic beating of CMs. On day 7, the amplitude sharply declined by over 53.4%, indicative of severe CM dysfunction resembling heart failure (Figure 6, “D” panel in the AngII/PE group), which contrasted with the healthy condition where frequency remained constant and amplitude increased (Figure 6, “H” panel). Treatment of the ischemic heart‐on‐a‐chip model with low concentrations of CHP‐Lip/Alam showed similar trends to untreated disease states in frequency and amplitude, albeit with slightly less pronounced changes, whilst CV‐related parameters showed significant fluctuations (Figure 6, “CHP1” panel in the AngII/PE group). Interestingly, only CHP2 group demonstrated statistically significant functional recovery, with an ≈0.53‐fold decrease in frequency and a 1.24‐fold increase in amplitude at Day 7 compared to Day 0 baseline. Additionally, there was a reduction in the CV of frequency and the CV of amplitude, indicating alleviation of arrhythmic beating and stabilization of Ca^2+^ transients (Figure 6, “CHP2” panel in the AngII/PE group). This suggests that administering CHP‐Lip/Alam above a specific concentration can restore the Ca^2+^ transients disrupted by the induced ischemia, thereby improving CM beating function. Furthermore, parameters such as 80% decay time and time to peak in CHP‐Lip/Alam‐treated conditions also exhibited different profiles compared to untreated diseased conditions (Figure S14, Supporting Information). These Ca^2+^ transient data of CM were consistent with the analysis in Figure 5, demonstrating that our ischemic heart‐on‐a‐chip platform provided a suitable model for evaluating drug‐induced recovery in CM beating function. In addition, longitudinal Ca^2+^ transient recordings across all conditions (Videos S3–S5), together with whole‐chip immunofluorescence imaging at 7 days post‐treatment (Figure S15, Supporting Information), confirmed long‐term functional integrity and preserved tissue architecture with cTnT‐positive CMs and CD31‐positive vasculature in our engineered heart‐on‐a‐chip model, while also capturing pathological features such as arrhythmic beating and vascular disruption under ischemic conditions.

## 3 Conclusion

This study established an advanced in vitro vascularized heart‐on‐a‐chip platform that encompasses a coculture of CMs, CFs, and CEs in direct physical contact, which can be used to model clinically relevant myocardial ischemia upon exposure to a combination of vasoconstrictors. Unlike existing heart disease models, our approach features perfusable cardiac microvessels within the engineered myocardial tissue, allowing for direct physical contact. This model thus provides essential physical and biochemical interactions for recapitulating a pathophysiologically relevant human cardiac tissue environment as well as facilitating the study of vascular disruption and associated environmental changes when mimicking heart disease progression. We found that healthy engineered myocardial tissue exposed to various vasoconstrictors exhibited characteristics associated with myocardial ischemia, such as arrhythmias, fibrosis, and vascular disruption. These findings validate the critical pathological effect of vasoconstrictors on myocardial tissues, thereby enhancing our understanding of the pathological mechanisms involved in developing effective heart‐remodeling therapies.

We also demonstrated the potential of our vascularized and perfusable heart‐on‐a‐chip model under induced‐ischemic conditions to analyze the therapeutic performance of drug‐loaded nanocarriers. CHP has been validated in previous studies to enhance the targeting ability of nanoparticles to ischemic cardiac tissue. Indeed, in our ischemic heart‐on‐a‐chip, CHP‐conjugated liposomes exhibited higher localization than ScP‐conjugated liposomes. Furthermore, when CHP‐Lip/Alam, known to have cardioprotective function in various heart diseases, was injected into the ischemic heart‐on‐a‐chip at various concentrations, we observed that several disease‐related markers were reversed. This included improved contractility, reduced fibrosis, and angiogenesis at the protein and mRNA levels. A real‐time Ca^2+^ transient analysis also highlighted functional recovery of myocardium after administration of CHP‐Lip/Alam at a specific concentration. To our knowledge, this is the first demonstration of using a vascularized heart‐on‐a‐chip model to analyze the targeting ability of nanomedicine and the performance of engineered cardiac tissue remodeling, highlighting the potential of this platform for modeling and evaluating nanotherapies in heart diseases.

For future work, this vascularized and perfusable heart disease model can be advanced by incorporating patient‐derived stem cells and genetically mutated stem cell‐differentiated myocardial tissue component cells^[^ ^53^ ^]^ to provide a patient‐specific disease modeling approach. Additionally, implementing decellularized ECM from diseased heart tissue^[^ ^54^ ^]^ or injecting relevant immune cells^[^ ^55^ ^]^ can further facilitate the construction of a more complex heart disease model that approaches clinical parity. Introducing variable durations of pathological stimulation may also enable the elucidation of temporally dynamic remodeling stages, thereby capturing transient adaptations that precede irreversible dysfunction. These models will enable a deeper exploration of the biological interface between the vasculature and myocardium, as well as the effects of external factors such as varying vascular flow shear rates and flow conditions, including thrombosis, in pathological conditions.^[^ ^56^ ^]^

We believe this technology will accelerate the efficiency of drug and nanocarrier development by enabling real‐time analysis of contractility function, nanocarrier targeting imaging, and drug efficacy under various disease conditions. Additionally, it holds the potential to construct patient‐specific in vitro heart models, promoting innovative opportunities for developing personalized therapies with minimized off‐target side effects.

## 4 Experimental Section

### 3D Human Vascularized and Perfusable Heart‐on‐a‐Chip Preparation

The 3D human vascularized and perfusable heart‐on‐a‐chip was generated based on a previously described protocol^[^ ^17b^ ^]^ with some modifications. A 6:5:1 cell ratio of hiPSC‐derived CMs, CEs, and CFs was used to reflect the overall volumetric ratio of CMs to non‐CMs in the mammalian heart.^[^ ^19^ ^]^ Myocardial tissue culture medium was prepared by mixing RB+ medium and EGM‐2MV medium in a 1:1 ratio and adding aprotinin (Sigma‐Aldrich, A3428) at a final concentration of 1 µg mL^−1^. On day 0, the stabilization medium was prepared by adding 10% (v/v) fetal bovine serum (FBS) and 10 nm Y‐27632 (STEMCELL Technologies, 72307) into the myocardial tissue culture medium. The cell mixture was suspended in a stabilization medium with 5 U mL^−1^ thrombin (T7201‐100UN, Sigma‐Aldrich). The cell mixture solution was further homogeneously mixed with a 10 mg mL^−1^ fibrinogen solution in a 1:1 ratio. This final solution (0.48 million cells/channel) was quickly injected into the middle channel in the microfluidic device (Aim Biotech, DAX‐1). After 15 min of gelation in the incubator at 37 °C with 5% CO_2_, the stabilization medium was added to the side channels. On day 1, the medium was replaced with a myocardial tissue culture medium. After 3–4 days, 10 µL of CE (8 million cells mL^−1^) was introduced into one side channel, and the chip was placed tilted for 30 min to encourage even distribution of cells during seeding on the side of gel in the central channel, before tilting the chip the other way for seeding homogeneity. These prepared chips were incubated at 37 °C with 5% CO_2_. The media was replaced through side channels via a hydrostatic pressure difference every 12 h, resulting in interstitial flow throughout the gel‐laden central channel. After 3–4 more days, a 3D vascularized and perfusable heart‐on‐a‐chip was prepared, exhibiting spontaneous heart beating. For early experiments characterizing AngII/PE‐induced pathophysiological changes (Figures 2 and 3), 3D vascularized myocardial tissues were also used in parallel under off‐chip conditions. The same gel composition and cell ratio were used, and the final cell‐fibrin mixture was seeded into high ibiTreat plates (80806, ibidi) instead of the microfluidic device. Although no further endothelial cell seeding was conducted in this format, the tissues were maintained in myocardial tissue culture medium under identical incubation and medium exchange protocols as those used in the chip‐based system.

### Myocardial Ischemia Induction

The engineered myocardial ischemia model was induced using a medium containing angiotensin II (AngII, Sigma‐Aldrich, A1153) and phenylephrine (PE, Sigma‐Aldrich, P6126). First, a stock solution containing 2 µm of AngII and 300 µm of PE was prepared, and serial dilutions were performed using myocardial tissue culture medium to obtain the final working concentrations for subsequent experiments. After confirming the quality of the 3D heart‐on‐a‐chip in terms of perfusion and heart beating, AngII/PE was added to the medium with different concentrations into the side channel, with a hydrostatic pressure difference, and replaced every 12 h.

### Spontaneous Beating Rate Measurement

To measure alterations in Ca^2+^ transients of 3D‐engineered human myocardial tissue and heart‐on‐a‐chip, systems consisting of CMs differentiated from hiPSCs containing GCaMP6f cassettes, CFs, and CEs were incubated in Tyrode's buffer (Alfa Aesar, J67607 AP), which replaced culture medium. For Figure 2, Ca^2+^ transients were measured in off‐chip 3D myocardial tissues, while Figure 6 used the vascularized and perfusable heart‐on‐a‐chip models. Platforms were transferred to a temperature‐regulated fluorescence microscope (Carl Zeiss, LSM 780) stage set at 37 °C and 5% CO_2_. Imaging spontaneous beating was performed using a wide‐field microscope with an emission filter setting (excitation: 495 nm, emission: 520 nm) for over 30 s at a 10‐millisecond image capture interval. For off‐chip 3D myocardial tissues, 3 ROIs per tissue were selected in the central area of the matrix. For heart‐on‐a‐chip models, two distinct regions were defined per myocardial channel, and 5 ROIs were selected per region (10 ROIs in total). The fluorescence intensity of each image frame over time was quantified using ImageJ software, and the acquired data was analyzed using in‐house‐developed MATLAB codes, enabling the computation of frequency, amplitude, time to peak, and 80% decay time of individual beats in each condition. Arrhythmicity was evaluated indirectly by examining the CV of beating frequency and amplitude, extracted from individual Ca^2+^ transient peaks. To trace 2D Ca^2+^ transients, “MBF” and “F div F0” plugins were performed, and the “Reslice” function was applied to a specific ROI using ImageJ software.

### Verification of Protein Expression Measurement with Immunofluorescence Imaging

After washing with DPBS thrice, 3D‐engineered myocardial tissues and heart‐on‐a‐chip were then fixed using 4% (w/v) paraformaldehyde (Electron Microscopy Sciences, #15710) for 30 min at room temperature, followed by three DPBS washes. Next, it was permeabilized using 0.1% (v/v) Triton X‐100 (Sigma‐Aldrich, X‐100) in DPBS for 30 min at room temperature. After washing thrice with DPBS, 5% (v/v) of FBS in DPBS was used to block the non‐specific binding of the antibody in the tissue for 1 h incubation at room temperature. Fluorescence‐labeled antibodies against cTnT (BD Bioscience, 565744, 1:400), Vimentin (Abcam, ab154207, 1:500), FAP‐α (Bioss Antibodies, BS‐5758R‐A555, 1:100), α‐SMA (Abcam, ab202295, 1:100), and Type I collagen (Bioss Antibodies, BS‐10423R‐A647, 1:100) were used for analysis of myocardial infarction‐related proteins. A 1:400 diluted anti‐CD31 antibody (Abcam, ab215911) was used as an endothelial cell marker. After overnight incubation at 4 °C, the cell nuclei were stained with 1 µM Hoechst 33342 (Merck, B2261) for 30 min and then washed three times with DPBS. Imaging for Figure 3 was performed on 3D myocardial tissues prepared off‐chip, while Figures 4 and 5 used samples from vascularized and perfusable heart‐on‐a‐chip models. The z‐stacked fluorescence images of stained samples were captured using a confocal microscope (SP8, Leica) with 40 µm thickness and 1 µm z‐spacing for 3D myocardial tissue and with 160 µm thickness and 5 µm z‐spacing for 3D heart‐on‐a‐chip models. ImageJ software was used for image processing and quantification of protein expression.

### 3D Vessel Morphology Analysis

After infusing 0.3/45 µm AngII/PE into the 3D vascularized heart‐on‐a‐chip for 7 days, the signal from Alexa Fluor 488‐tagged CD‐31 antibody‐stained CEs was used to quantify vascular area percentage, vascular network length, and average vessel diameter using ImageJ.^[^ ^57^ ^]^ The samples were fixed on day 14, and z‐stacked confocal images were acquired with 150 µm thickness and 5 µm z‐spacing using a confocal microscope (SP8, Leica). 3 ROIs per chip were selected randomly. Maximum intensity projections were generated from z‐stacked images. Following brightness and contrast adjustments, the noise was filtered by applying a “Gaussian blur 3D” function with a 2.0 value of x, y, and z sigma. Images within each ROI were then converted to a binary format using a triangle threshold adjustment. Artifacts were then removed through the “erode” and “remove outliers” functions. The vessel area was quantified using the “measure” function in binary images. After the “2D skeletonize” and “analyze skeleton (2D/3D)” functions were employed, the total vessel network length, number of junctions, and number of vessel endpoints were quantified. The average vessel diameter was calculated by dividing the total vessel area by the total vessel network length. Connectivity was calculated as the ratio of the number of vessel junctions to the number of vessel endpoints. All image processing and analyses were performed using plugins in ImageJ software.

### Preparation of Cardiac Homing Peptide‐Conjugated Liposome Loading Alamandine

Alamandine‐loaded liposomes were produced by thin film hydration followed by extrusion. A mixture of 1,2‐distearoyl‐sn‐glycero‐3‐phosphocholine (24 mg, DSPC, Sigma‐Aldrich, 850365P), cholesterol (12 mg, Sigma‐Aldrich, C8667), and 1,2‐distearoyl‐sn‐glycero‐3‐phosphoethanolamine‐N‐[maleimide(polyethylene glycol)‐2000] (1 mg, DSPE‐PEG‐Maleimide, Sigma‐Aldrich, 880126P) was dissolved in 500 µL of a 9:1 chloroform/methanol solution. The solution was dried under a stream of N_2_, followed by vacuum drying overnight to form the film. The film was rehydrated with an 8 mm solution of Alamandine (Insight Biotechnology, HY‐P3108) in 500 µL DPBS and stirred at 40 °C for 30 min. Solutions were subjected to freeze‐thaw by freezing at ‐80 °C and thawing at 40 °C repeatedly, and after 4∼5 freeze‐thaw cycles, a homogenous turbid solution was achieved after vortexing. Liposomes were then extruded sequentially through 200, 100, and 50 nm porous membranes (Cytiva, 10417004, 10419504, and 800308, respectively) using an Avanti mini extruder (Avanti Polar Lipids, 610000), passing through each membrane 21 times. For DiD‐labeled liposome preparation, the same protocol was followed, adding 120 µL of 1 mg mL^−1^ DiD solution (ThermoFisher Scientific, V22887) to the initial liposome mixture in a 9:1 chloroform/methanol solution. To make peptide‐conjugated liposomes, a 20:1 molar ratio of tris(2‐carboxyethyl)phosphine (TCEP, ThermoFisher Scientific, T2556) to peptide was made up in DPBS and added to the liposomes (1:1 molar ratio of maleimide to peptide). Peptides were custom‐synthesized by Elim Biopharm with the sequence CSTSMLKAC for CHP and CSKTALSMC for the ScP. The ScP is chemically identical but has a randomized internal sequence. Samples were stirred at room temperature for 6 h and then placed in the fridge at 4 °C overnight. The liposome solutions were purified using size exclusion chromatography to remove excess drug, dye, and free peptide by running through PD MiniTrap G‐25 columns (Cytiva, 28‐9180‐07) in DPBS twice sequentially. Peptide‐conjugated liposomes containing Alamandine were stored at 4 °C for further experiments. Further details about characterization can be found in the supplementary information.

### Data and Statistical Analysis

The experimental data were normalized by Excel and processed by Matlab for spontaneous beating rates. Data were plotted as mean ± standard deviation of the mean using GraphPad Prism 8. After the normality test, two‐tailed unpaired Student's t‐test, one‐way or two‐way ANOVA, or a non‐parametric test, the Kruskal‐Wallis test, was used to compare among groups. For all comparisons, the p‐value with a significant difference was indicated as follows (*p < 0.05; **p < 0.01; ***p < 0.001; ****p < 0.0001).

## Conflict of Interest

M.M.S. has invested in, consults for (or is on scientific advisory boards or boards of directors) and conducts sponsored research funded by companies related to the biomaterials field; has filed patent applications related to biomaterials; and has co‐founded companies in the biomaterials field. The rest of the authors declare no conflict of interest.

## Author Contributions

J.K. and X.Z. contributed equally to this work. J.K. and M.M.S. designed the research. J.K., X.Z., A.N., Q.Y.L., A.C.‐N., Y.K.K., R.X., and H.K. conducted the experiments. J.K., X.Z., R.W., K.X., J.A.K., T.‐E.P., H.L., and J.J. contributed to data analysis and provided conceptual advice. All authors discussed the results and contributed to the preparation of the paper. J.K. drafted the manuscript. X.Z., R.W., A.N., Q.Y.L., A.C.‐N., J.A.K., Y.K.K., K.X., R.X., H.K., H.L., T.‐E.P., J.J., and M.M.S. revised the paper. M.M.S. supervised the study. All authors approved the final version of the manuscript.

## Supporting information



Supporting Information

Supporting Information

Supporting Information

Supporting Information

Supporting Information

Supporting Information

## Data Availability

The data that support the findings of this study are available from the corresponding author upon reasonable request.
