# Supplementary material for: Vascularized and Perfusable Human Heart‐on‐a‐Chip Model Recapitulates Aspects of Myocardial Ischemia and Enables Analysis of Nanomedicine Delivery
Source: Adv Mater. 2025 Jul 26;37(41):e18909. doi: 10.1002/adma.202418909 (PMC12531757; doi:10.1002/adma.202418909)
Supplement: Supplementary file 1 — Supporting Information [file ADMA-37-e18909-s005.docx]

Supporting Information

Vascularized and Perfusable Human Heart-on-a-Chip Model Recapitulates Aspects of Myocardial Ischemia and Enables Analysis of Nanomedicine Delivery

*Junyoung Kim^†1,2^, Xuening Zhang^†1^, Richard Wang^1^, Adrian Najer^1^, Qiao You Lau^1,2^, Ana Cammack-Najera^1,2^, Jang Ah Kim^1^, Yoo Kyung Kang^1^, Ruoxiao Xie^1^, Hyemin Kim^1^, Kai Xie^1,2^, Tae-Eun Park^3^, Hyeonji Lim^3^, Jinmyoung Joo^3,4,5^, and Molly M. Stevens^1,2^**

**Experimental Methods**

*Cell Culture*: Human iPSC line (WTC-11, hiPSCs) and GCamP hiPSC line (hiPSCs containing GCaMP6f cassette) were generously provided by Professor Bruce Conklin in Gladstone Institue, USA. These lines were generated from a healthy male donor who signed a consent form approving the donation of iPSCs to the public stem cell bank of the Coriell Institue. They were maintained in a complete Essential 8™ medium (ThermoFisher Scientific, A1517001) on 6-well plates coated with Cultrex (R&D Systems, 3433-010-01) diluted in DMEM/F12 medium (ThermoFisher Scientific, 11320033). For the initial 24 hours after passage, the cells were cultured in a complete Essential 8™ medium with 10 µM Y-27632 (STEMCELL Technologies, 72307) to improve survival after cell dissociation. The cells were routinely passaged every 3 days with a 1:12 split ratio. Human cardiac fibroblasts (CF, PromoCell, C-12375) were cultured in Fibroblast growth medium (PromoCell, C-23025) and cardiac human microvascular endothelial cells (CEs, Lonza, CC-7030) were cultured in Microvascular Endothelial Cell Growth Medium-2 (EGM-2MV) bullet kit (Lonza, CC-4147). CFs and CEs were used before passage 6, and the cell confluency was maintained at around 50~90%. The medium was changed every other day. All cells were cultured in the incubator at 37 °C with 5% CO_2_.

*Cardiomyocyte Differentiation*: hiPSC differentiation into CMs was performed based on a previously reported protocol ^[1]^. When hiPSCs reached approximately 90% confluence, the medium was changed to RPMI (Gibco, 11875-093) supplemented with 2% (v/v) B27 insulin (-) supplement (Gibco, 1895601) and 6 µM CHIR99021 (Tebu-bio, 04-0004-02). After 2 days of culture, the RPMI supplemented with 2% (v/v) B27 insulin (-) supplement (RB- medium) was replaced. After 1 day of culture, the medium was replaced with RB- medium with 2.5 µM Wnt-C59 (Stratech, S7037-SEL). After 2 days of culture, the medium was replaced with RB- medium. After 1 day of culture, the medium was replaced with RPMI supplemented with 2% (v/v) B27 supplement (ThermoFisher Scientific, 17504044) (RB+ medium) and was refreshed every other day until spontaneous contraction of the cells was exhibited. Metabolic selection of CMs was performed with RPMI without glucose (Gibco, 11879-020) supplemented with 2% (v/v) B27 supplement (ThermoFisher Scientific, 17504044) and 5 mM sodium lactate (Sigama-Aldrich, L4263) from day 11 to day 17, with an every other day exchange for purification of non-differentiated cells. After metabolic selection, the differentiated CMs were dissociated using collagenase type II (Thermo Fisher Scientific, 17101-015) for 3 hours and replated onto a Cultrex (R&D Systems, 3433-010-01)-coated plate for further experiments. The CMs were maintained in an RB+ medium with every other day exchange. The same protocol was used to differentiate GCaMP hiPSCs from CMs. To evaluate the CM differentiation, detached CMs were fixed with 4% (w/v) paraformaldehyde (Electron Microscopy Sciences, #15710) for 10 minutes, followed by permeabilization using 0.1% (v/v) Triton-X100 (Sigma-Aldrich, X-100) for 10 minutes and stained with Alexa Fluor® 647 isotype antibody (BD Bioscience, 557732) as control and Alexa Fluor® 647 mouse anti-cTnT (BD Bioscience, 565744), diluted with 1:400 in a 2% (v/v) fetal bovine serum (FBS) solution in Dulbecco’s Phosphate Buffered Saline (DPBS) for overnight at 4 °C. Cells were washed with DPBS with 2% (v/v) FBS between each step. hiPSCs were used as a negative control for comparison with the same flow cytometry sample preparation. Finally, the stained cells were analyzed using flow cytometry (BD Biosciences, LSRFortessa^TM^), and the data were processed by FlowJo software v10.8.1(BD Biosciences).

*Measurement of Hypoxia Level of 3D-Engineered Myocardial Tissue on a Microfluidic Platform*: The preparation of 3D-engineered myocardial tissue models on a microfluidic platform followed the protocol for creating 3D human vascularized and perfusable heart-on-a-chip described in the main text. For the cardiomyocytes-only conditions, hiPSC-derived CMs were used exclusively, replacing other supporting cell types. In the vascularized myocardial tissue condition, the step of introducing cardiac endothelial cells into side channels was omitted. The models were cultured using the same myocardial tissue culture medium. The prepared chips were incubated at 37 °C with 5% CO_2_. The media was replaced every 12 hours. To assess hypoxia levels, the microfluidic platforms were incubated with a 1:1000 dilution of a hypoxia detection reagent stock solution (ENZ-51042, Enzo Life Sciences) for 1 hour. The detection reagent is based on a fluorescence mechanism involving nitroreductase activity in hypoxic cells, which reduces a nitroaromatic compound to emit fluorescence. After incubation, the samples were washed with PBS and imaged using a confocal microscope (SP8, Leica) with excitation at 490 nm and emission at 525 nm. Z-stacked fluorescence images were acquired, and ImageJ software was used for image processing and quantification of hypoxia-related fluorescence signals.

*Cytotoxicity Measurement using CCK-8*: Cytotoxicity was measured using the cell counting kit-8 (CCK-8) (Sigma-Aldrich, 96992). The cells (CM, CF, and CE) were seeded separately with a density of 5,000 cells per well in the Cultrex (R&D Systems, 3433-010-01, 1:100 diluted with DMEM/F12 medium)-coated 96-well plates. The viability of cells was measured after treatment with different concentrations of AngII/PE from 1 day to 3 days or with different concentrations of CHP-Lip/Alam for 1 day. The cell medium was changed every day. On the measurement day, 10 μL CCK-8 solution was added and incubated for 3 hours at 37 °C with 5% CO_2_. The absorbances were measured at 450 nm and 650 nm using the UV-vis spectrophotometer (SpectraMax M5, Molecular Devices), and cell viability was calculated.

*Excitation Threshold Measurement*: The excitation threshold of 3D-engineered human myocardial tissue was determined as the ability to follow 2 Hz electrical field stimulation, which is higher than the frequency of spontaneous heartbeats in all conditions and necessary to prevent inducing arrhythmia ^[2]^. The myocardial tissue culture chamber was connected to the stimulator (MYP100, IonOptx) using platinum electrodes placed at fixed positions on opposite ends, delivering a uniform electric field across the entire engineered 3D myocardial tissue. The stimulation voltage began at 1 mV and increased in 0.5 mV increments until externally paced contractions matched the electrical stimulation frequency. To ensure consistent pacing of the engineered myocardial tissue, the final stimulation voltage was set at 0.5 mV above the excitation threshold. Beating behavior was acquired using the same imaging setup as that used for spontaneous beating rate measurement.

*Young’s Modulus Measurement of 3D Myocardial Tissue*: The 4 mm x 4 mm-sized 3D-engineered myocardial tissues with ~500 μm thickness were prepared on the PDMS structure (off-chip) for AFM measurements. After the replacement of myocardial tissue culture medium into Tyrode’s buffer (Alfa Aesar, J67607 AP), Young’s modulus of 3D myocardial tissue was measured using Atomic Force Microscopy (AFM, BlueScientific, JPK CellHesion 200) with a cantilever (Bruker AFM Probe, MLCT-016) functionalized with 25 μm radius spherical polystyrene beads having an average spring constant of 0.09 N/m and frequency of 38 kHz. The actual stiffness of the cantilever was calibrated in Tyrode’s buffer (Alfa Aesar, J67607 AP), and then measurements were performed with an indentation depth of ~3 μm using a constant probe speed of 5 μm/s. Young’s modulus was calculated from load-indentation curves by fitting to the Hertz model and assuming a Poisson’s ratio of 0.5 with a manufacturer’s data analysis software (JPK Instrument, JPK DP software). 10 x 10 grids in 100 μm x 100 μm maps were performed with 3 samples in each condition.

*Permeability Measurements*: The permeability of a 3D vascularized heart-on-a-chip with different conditions on day 14 was measured with 10 μg/mL of 70 kDa CF®633-tagged dextran (Biotium, 80141) in myocardial tissue culture medium. After injecting the solution using a pressure gradient and establishing equilibrium of pressure to prevent convection, confocal z-stacked and time-lapse images were acquired with a 200 μm thickness and a 10 μm z-spacing for 15 minutes at 1-minute intervals. Permeability was evaluated using the previously described protocols ^[3]^ followed by Stack, Threshold, Erosion, and Trainable Weka Segmentation 3D functions plugins in ImageJ. With acquired values about geometry and fluorescence intensity in the matrix and vessel over time, the permeability in each time point can be calculated by the below equations: $P (cm/s) =\frac{1}{\Delta t}\frac{V_{m}}{SA_{v}}\frac{\Delta I_{m}}{\Delta I}$, where Δt is the time between time points, V_m_ is the gel matrix volume, SA_v_ is the surface area of the vasculature, ΔIm = I_m2_ – I_m1_ is the change of average fluorescence intensity in the gel matrix, and ΔI = I_v1_ – I_m1_ is the difference in average intensity between the vasculature and matrix at the start point.

*Characterization of Cardiac Homing Peptide-Conjugated Liposome Loading Alamandine*: The size and zeta potential of peptide-conjugated liposome containing Alamandine were measured by DLS measurements using a Malvern Zetasizer Nano-ZS. FITC-labeled CHP and ScP (Molecular Dimensions) conjugated liposomes were used to confirm the conjugation of peptides with liposomes, which was made by the same peptide conjugation protocol. FITC fluorescence intensity of each liposome at 2 mg/mL concentration was measured at 495 nm emission and 515 nm excitation using a UV-vis spectrophotometer (SpectraMax M5, Molecular Devices). To measure the loading efficiency of Alamandine, 1 mg of lyophilized liposomes was physically dissolved using a tip sonicator (VCX 500, Sonics & Materials, Inc.) with a 20% amplitude for 1 minute and then filtered using a 3 kDa Amicon centrifugal filter (UFC900308, Merck Millipore). The concentration of Alamandine was determined by the absorbance using a UV-vis spectrophotometer (SpectraMax M5, Molecular Devices) at 220 nm wavelength.

*Liposome Administration into 3D Vascularized Heart-on-a-Chip for Evaluation of Liposome Targeting Ability and Cardiac Remodeling Efficacy*: To quantify liposome targeting ability, 300 μg/mL of DiD-stained and peptide-conjugated liposomes were diluted in a myocardial tissue culture medium. 160 μL and 80 μL of liposome solution were administered through each side channel to create different hydrostatic pressures for 30 minutes, with resets every 10 minutes, resulting in interstitial flow throughout the 3D myocardial tissue ^[4]^. After every cycle for 10 minutes, unattached liposomes were collected and washed with a myocardial tissue culture medium for 10 minutes. The z-stacked fluorescence images of localized DiD-stained liposomes were captured using a confocal microscope (SP8, Leica) with 130~140 μm thickness and 2 μm z-spacing after every administration of DiD-stained liposomes. To evaluate the distribution of DiD-stained liposomes within the vascular network of the myocardial tissue channel in the heart-on-a-chip, the chips were fixed with 4% (w/v) paraformaldehyde, permeabilized using 0.1% (v/v) Triton X-100, and blocked with 5% (v/v) FBS. The vascular network was stained with an anti-CD31 antibody (Abcam, ab215911), followed by z-stacked fluorescence imaging using a confocal microscope. Colocalization between DiD-labeled liposomes and CD31-positive vessels was analyzed using the JACoP plugin in Image J. Mander’s colocalization coefficient was calculated to quantify the degree of liposome localization within the vascular structures.

To determine the cardiac remodeling efficacy of liposomes, 250 μg/mL and 500 μg/mL of peptide-conjugated liposomes containing Alamandine were diluted in myocardial tissue culture medium with 0.3/45 µM AngII/PE. 160 μL and 80 μL of liposome solution were administered through side channels with different hydrostatic pressures for 3 hours, and the process was repeated every 10 minutes. Unattached liposomes were washed three times with myocardial tissue culture medium containing 0.3/45 µM AngII/PE for 10 minutes. This administration was performed every day. After 24 hours of incubation for each administration, the spontaneous beating rate was measured in Tyrode’s buffer, followed by the above protocol. Immunofluorescence imaging of FAP-α, Type I Collagen, and cTnT was also performed after 48 hours of peptide-conjugated liposomes containing Alamandine administration according to the above protocol.

*Reverse Transcription Quantitative PCR (RT-qPCR)*: To compare the relative gene expression levels between the groups, cells were first extracted from the microfluidic platform (pooled from 3 chips, *n* = 4 independent experiments) by adding 0.7 mg/mL collagenase type II (ThermoFisher Scientific, 17101015) for 30 minutes at 37 °C and centrifugation with 200 g for 5 minutes. Total RNA was collected from each sample using the Quick-RNA™ MicroPrep Kit (Zymo Research, R1050). Complementary deoxyribonucleic acid (cDNA) was synthesized using the cDNA synthesis kit (ThermoFisher Scientific, 4387406) following the manufacturer’s instructions. The mRNA level was determined by quantitative real-time PCR using TaqMan gene expression assays (FAM) and TaqMan Fast Advanced PCR Master Mix (ThermoFisher Scientific, 4444557) on QuantStudio™ 6 Flex Real-Time PCR System (Applied Biosystems). The primers used for the qPCR analysis are listed in the Supplementary information (**Supplementary Table 1**). The expression levels of specific genes were normalized to those of the endogenous reference GAPDH, and the relative gene expression levels were calculated using the comparative C_t_ method.

*Brain Natriuretic Peptide (BNP) Secretion Analysis*: Following the manufacturer’s instructions, the secreted BNP in the media was measured using the Human BNP ELISA kit (Abcam, ab193694). Medium was collected from vascularized heart-on-a-chip under different conditions on days 0, 4, and 7 of treatment. Cellular debris was removed by centrifugation at 1,000 g for 10 minutes at 4 °C. The supernatant was collected and stored frozen at -80 °C until measurement.

*Cardiomyocyte Size Measurement*: Cells were collected from the microfluidic platform using 0.7 mg/mL collagenase type II (ThermoFisher Scientific, 17101015) treatment and then washed twice with centrifugation at 250 g for 3 minutes. The collected cells were suspended in 4% (w/v) paraformaldehyde (Electron Microscopy Sciences, #15710) for 10 minutes, followed by permeabilization using 0.1% (v/v) Triton-X100 (Sigma-Aldrich, X-100) for 10 minutes and stained with Alexa Fluor® 647 mouse anti-cTnT (BD Bioscience, 565744) diluted with 1:400 in a 2% (v/v) FBS solution in DPBS for overnight at 4 °C. Cells were washed with DPBS with 2% (v/v) FBS between each step. The size calibration beads (Thermo Fisher Scientific, F13838) and stained cells were analyzed using flow cytometry (BD Biosciences, LSRFortessa^TM^). First, distinctive forward scatter (FSC) values were obtained to plot a calibration curve by beads with different sizes (10 μm and 15 μm). To determine the FSC values of cell samples using flow cytometry, CMs were gated with cTnT-positive cells after excluding debris and clumps. FSC values of these gated cells were converted to the size of CMs based on the calibration data from size calibration beads. This data was processed by FlowJo software.

**Figures**


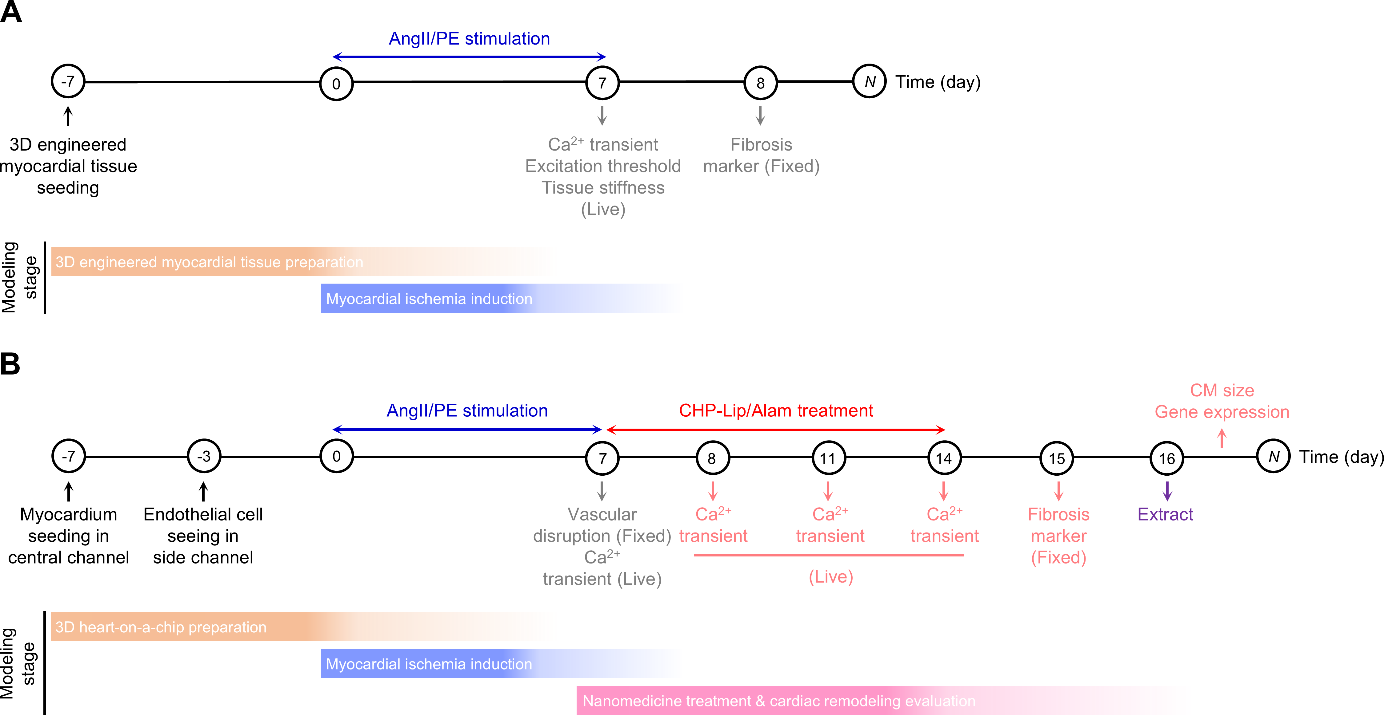


**Supplementary Figure 1. Schematic timeline summarizing the experimental workflow for myocardial ischemia modeling and therapeutic evaluation in 3D engineered human myocardial tissue models.** (A) Timeline for myocardial ischemia induction in 3D engineered myocardial tissue, including vasoconstrictor (AngII/PE) stimulation and corresponding assessment time points for Ca^2+^ transient analysis, excitation threshold, and tissue stiffness measurements in live tissues, followed by immunofluorescence-based fibrosis marker analysis in fixed tissues. (B) Timeline of nanomedicine (CHP-Lip/Alam) treatment and cardiac remodeling evaluation in the vascularized and perfusable heart-on-a-chip model. The schematic outlines the sequential steps of chip preparation, myocardial ischemia induction, nanomedicine infusion, and endpoint analyses, including Ca^2+^ transient, fibrosis marker, vascular disruption, gene expression profiling, and hypertrophy.


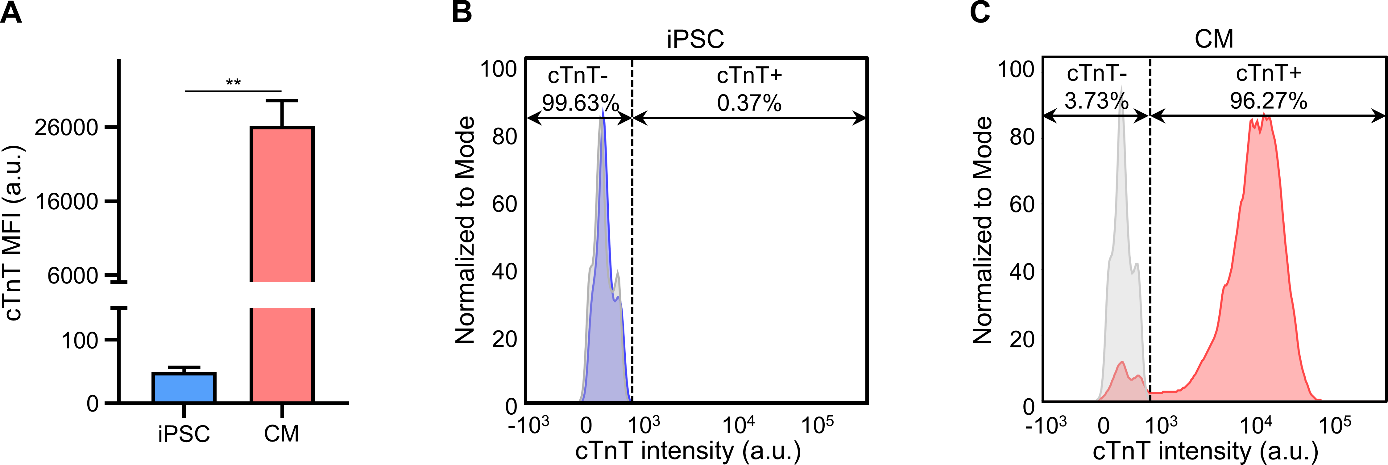


**Supplementary Figure 2. Flow cytometric analysis of cardiac troponin (cTnT) expression in hiPSCs and differentiated CMs.** (A) Mean immunofluorescence intensity of cTnT in iPSCs and CMs (*n* = 3 independent samples). (B-C) Histogram overlay showing isotype control goat IgG-Alexa Fluor® 647 (Gray) and cTnT-Alexa Fluor® 647 for iPSCs (blue) and CMs (red). The percentage of cTnT-positive cells (cTnT+) is the mean from *n* = 3. Two-tailed unpaired Student’s t-test was used to compare the two groups; ***, *p* < 0.001.


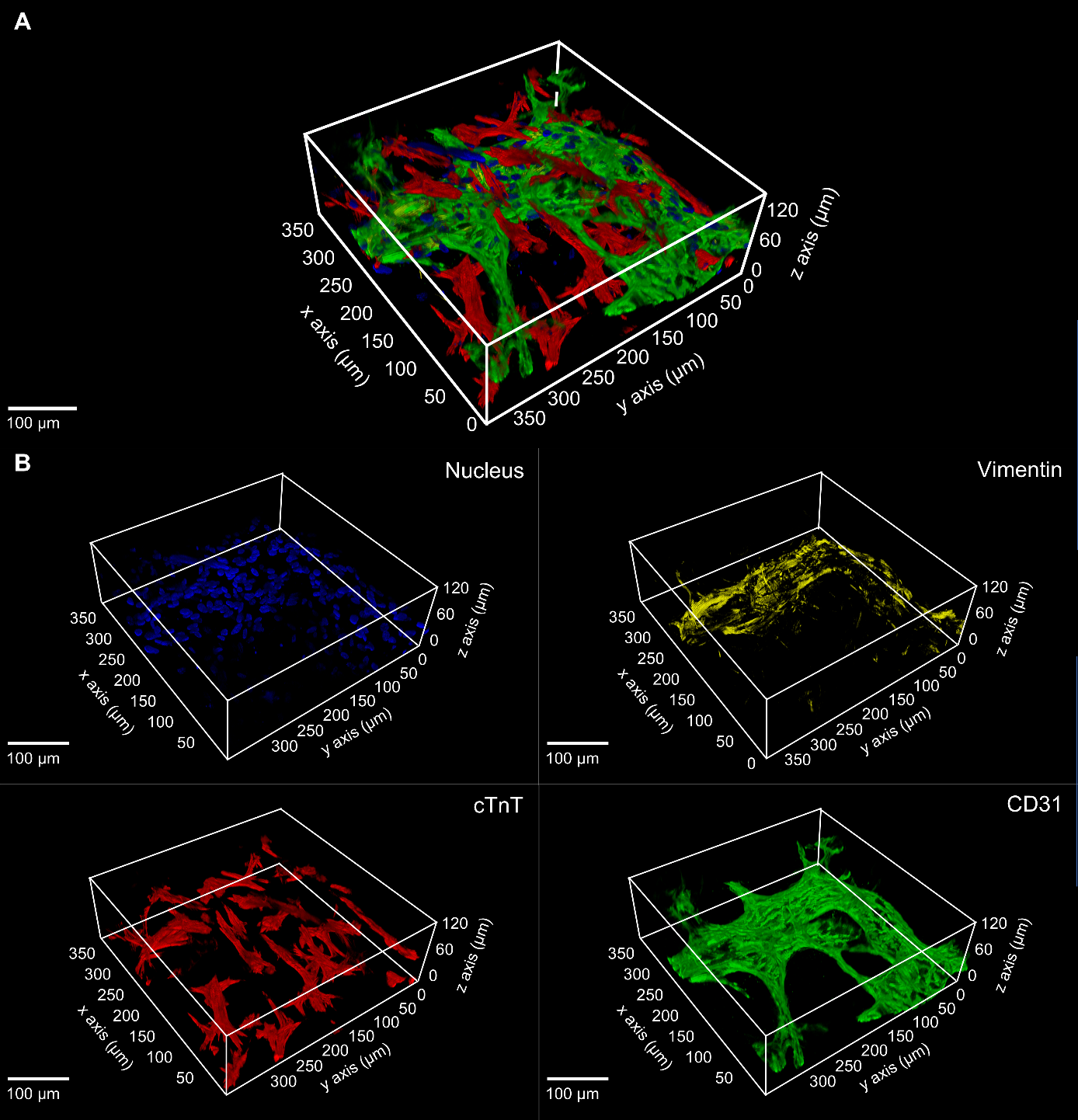


**Supplementary Figure 3**. 3D reconstruction of vascularized and perfusable heart-on-a-chip. (A) Merged 3D z-stack confocal image showing the spatial organization of nuclei (blue, Hoechst), cardiomyocytes (red, cTnT), cardiac fibroblasts (yellow, vimentin), and endothelial cells (green, CD31). (B) Individual 3D channel views of each component demonstrate distinct localization of cell types: nuclei (Hoechst), vimentin-positive fibroblasts, cTnT-positive cardiomyocytes, and CD31-positive endothelial cells. The engineered tissue exhibits a thickness ranging from 120 to 150 µm, as determined by z-axis reconstruction. Scale bars: 100 µm.


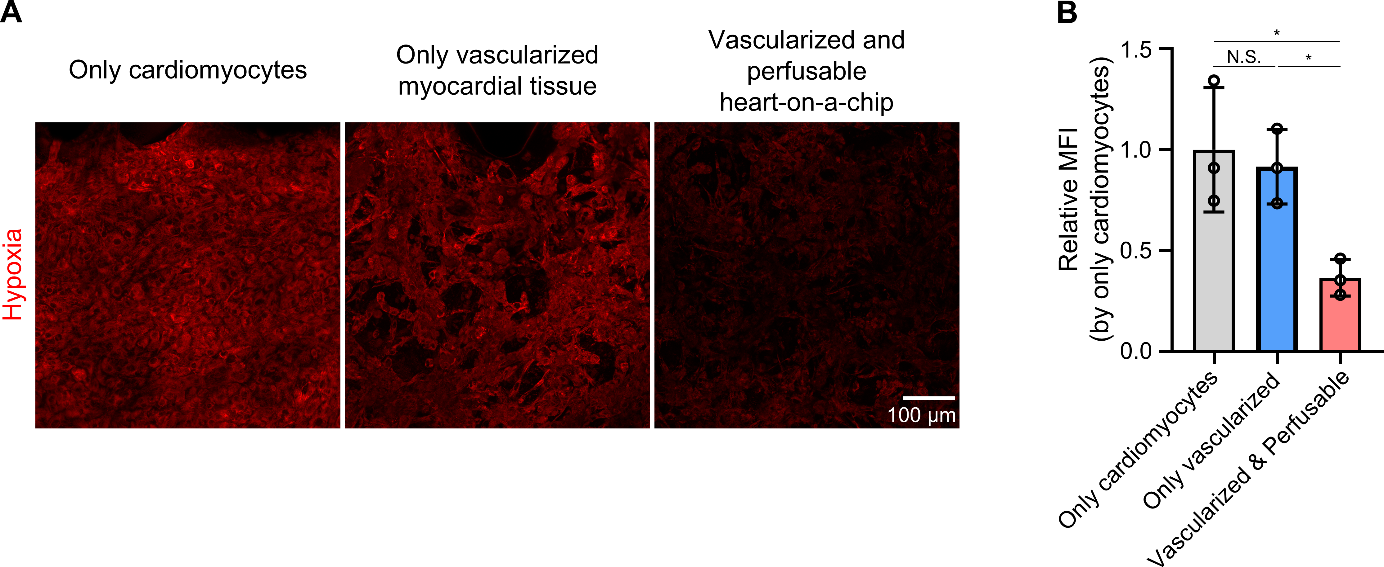


**Supplementary Figure 4**. Assessment of hypoxia levels to evaluate the physiological impact of vascular perfusion in a 3D heart-on-a-chip platform. (A) Representative z-stacked immunofluorescence confocal images (max. intensity projections) showing hypoxia levels (red) in engineered myocardial tissue in a microfluidic platform under three conditions: only cardiomyocytes (left), vascularized but non-perfusable myocardial tissue model (middle), and vascularized and perfusable heart-on-a-chip model (right). Scale bar: 100 µm. (B) Quantification of relative mean fluorescence intensity (MFI) of hypoxia signals normalized to the cardiomyocyte-only condition. Each point represents data from 1 chip. Total of 3 independent chips. One-way ANOVA with post hoc Tukey’s test was used to compare the differences among conditions after the normality test; N.S., not significant; *, p < 0.05.


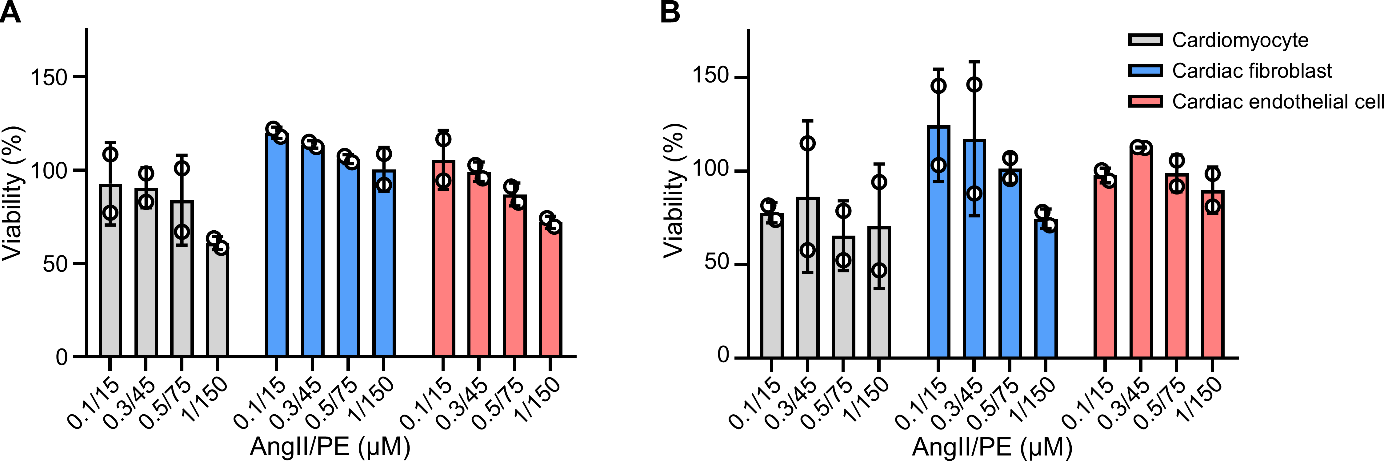


**Supplementary Figure 5. Cytotoxicity of different concentrations of vasoconstrictors on different cell types of the 3D vascularized heart-on-a-chip.** (A-B) CCK-8 assay of cell viability of cardiomyocytes, cardiac fibroblasts, and cardiac endothelial cells incubated with different concentrations of vasoconstrictors (AngII 0.1 µM–1.0 µM and PE 15 µM–150 µM) at Day 1 (A) and Day 3(B) (4 technical measurements from *n* = 2 independent samples).


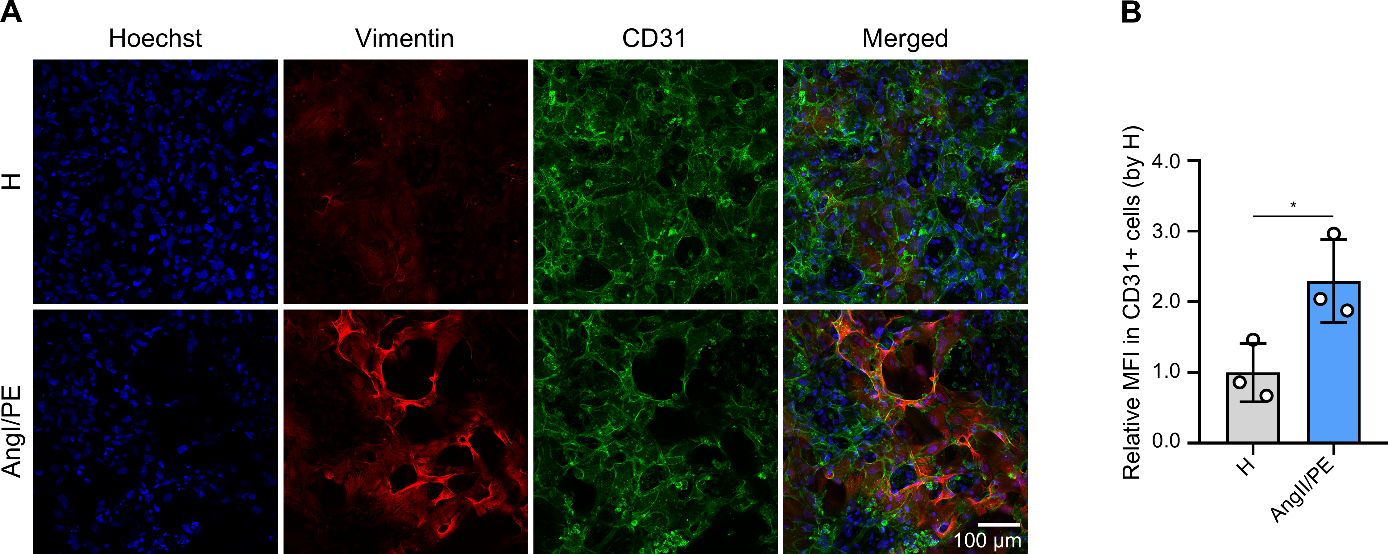


**Supplementary Figure 6. Endothelial-to-mesenchymal transition (EndMT) induced by AngII/PE infusion in vascularized heart-on-a-chip models.** (A) Representative z-stacked immunofluorescence confocal images (max. intensity projection) showing vimentin (red), CD31 (green), and nuclei (blue) in healthy (H) 3D heart-on-a-chip models and models treated with 0.3/45 µM AngII/PE. Scale bar: 100 µm. (B) Quantification of vimentin expression in CD31^+^ cells from healthy and 0.3/45 µM AngII/PE-treated heart-on-a-chip models. Each point represents data from one independent chip. The two-tailed unpaired Student’s t-test was used to compare the means of the two groups; *, *p* < 0.05.


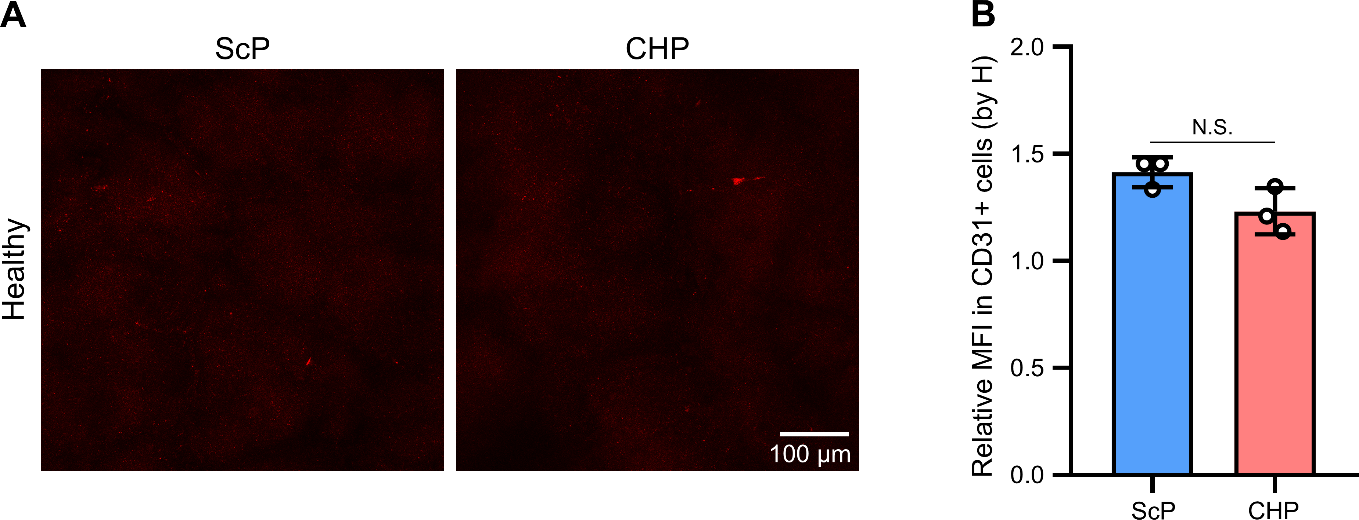


**Supplementary Figure 7. Comparison of the accumulation of liposomes conjugated with scrambled peptides (ScP) and cardiac homing peptides (CHP) in a 3D healthy vascularized heart-on-a-chip.** (A) Representative confocal images of accumulated liposomes (red) after 3 cycles of infusion in 3D healthy vascularized heart-on-a-chip tissues. Scale bar: 100 µm. (B) Quantification of fluorescence intensity of accumulated liposomes in the 3D healthy vascularized heart-on-a-chip. Each point represents data from the average of 3 ROI in one chip. Total *n* = 3 independent chips. The two-tailed unpaired Student’s t-test was used to compare the means of the two groups; N.S., not significant.


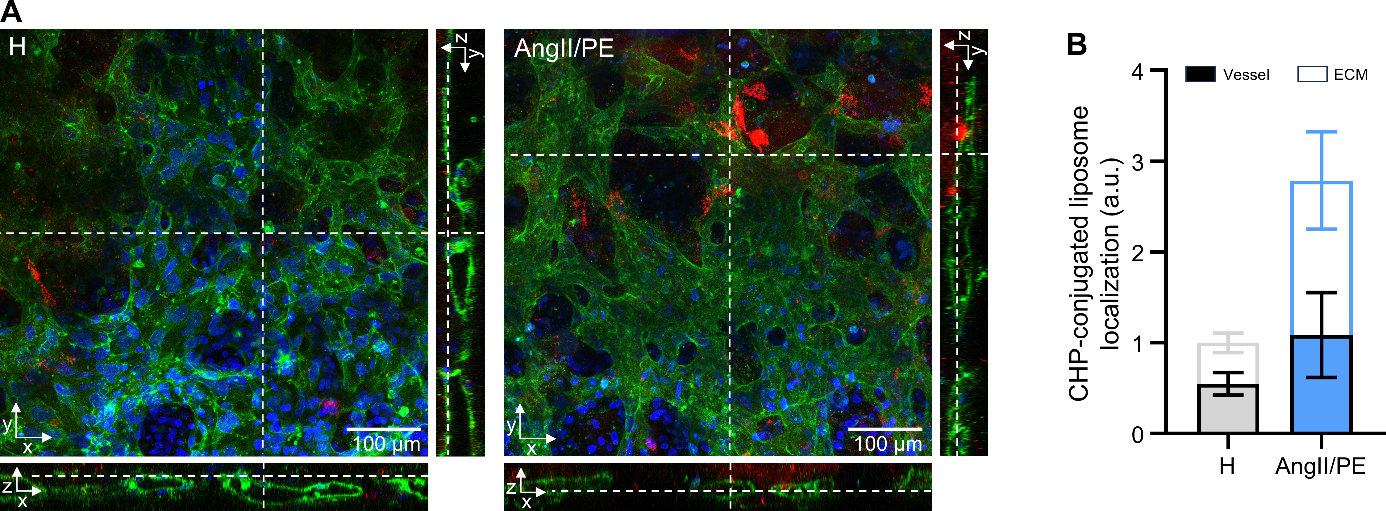


**Supplementary Figure 8. Distribution of CHP-conjugated liposomes in healthy and AngII/PE-treated heart-on-a-chip models.** (A) Representative orthogonal confocal images (z-stack projections) showing the distribution of DiD-labeled CHP-conjugated liposomes (red) in the vascularized myocardial tissue channel (green: CD31-positive vessels; blue: nuclei). Scale bar: 100 µm. (B) Quantification of liposome distribution, presented as the proportion of DiD signal localized within vessel regions versus extravascular extracellular matrix (ECM), under healthy (H) and AngII/PE-treated ischemic conditions. Plot represents mean ± standard deviation from 3 independent chips, normalized to total DiD intensity under healthy conditions.


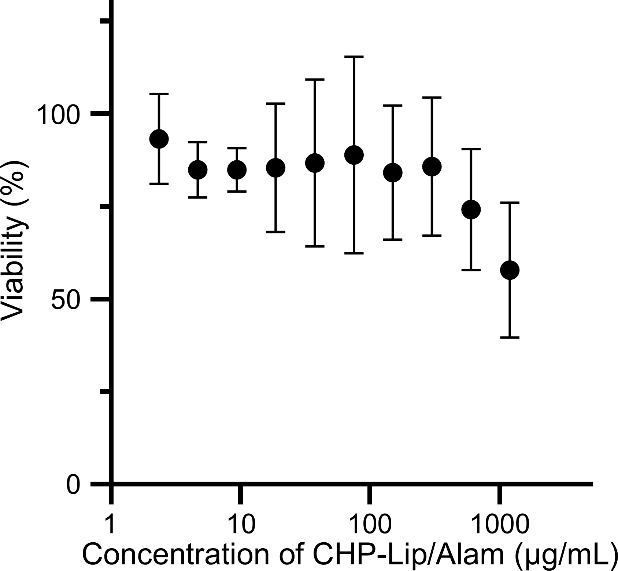


**Supplementary Figure 9. Cytotoxicity of different concentrations of CHP-Lip/Alam on hiPSC-derived CMs in 96 well plates.** CCK-8 assay of cell viability of CMs incubated with different concentrations of CHP-Lip/Alam after 24-hour treatment (6 technical measurements from 3 independent samples).


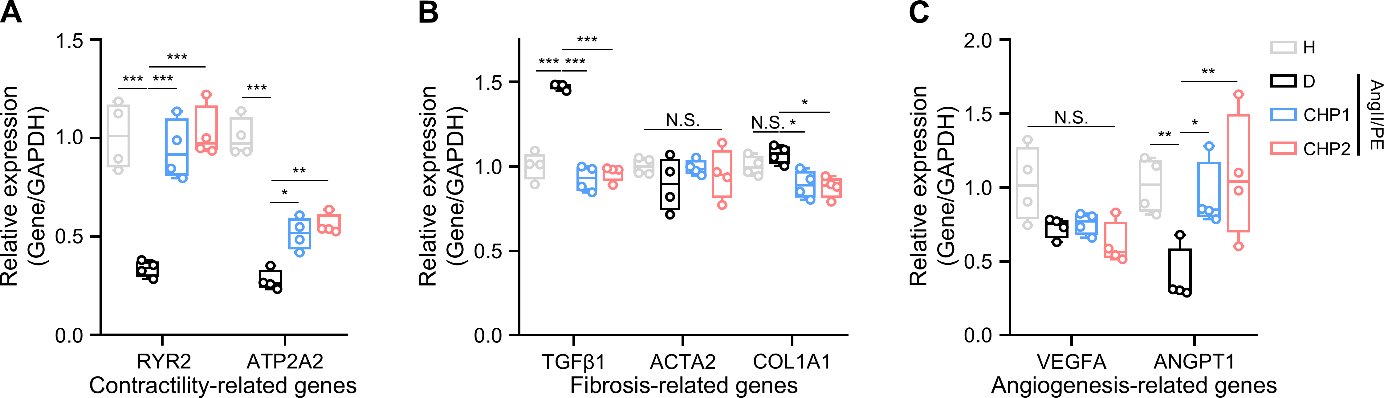


**Supplementary Figure 10. Box graph of the fold changes of contractility, fibrosis, and angiogenesis-related genes from reverse transcription quantitative PCR (RT-qPCR).** (A) Contractility-related genes. (B) Fibrosis-related genes. (C) Angiogenesis-related genes. Each point represents data from each sample, which was prepared by pooling *n* = 3 chips and *n* = 4 independent experiments; Two-way ANOVA with post hoc Tukey’s test were used to compare the difference of each condition after the normality test; N.S., not significant; *, *p* < 0.05; **, *p* < 0.01; ***, *p* < 0.001. The box plot represents the central line, denoting the median value, while the box contains the 25th to 75th percentiles of the dataset, with whiskers marking the maximum and minimum values. This data was represented as a heatmap in **Figure 5L**.


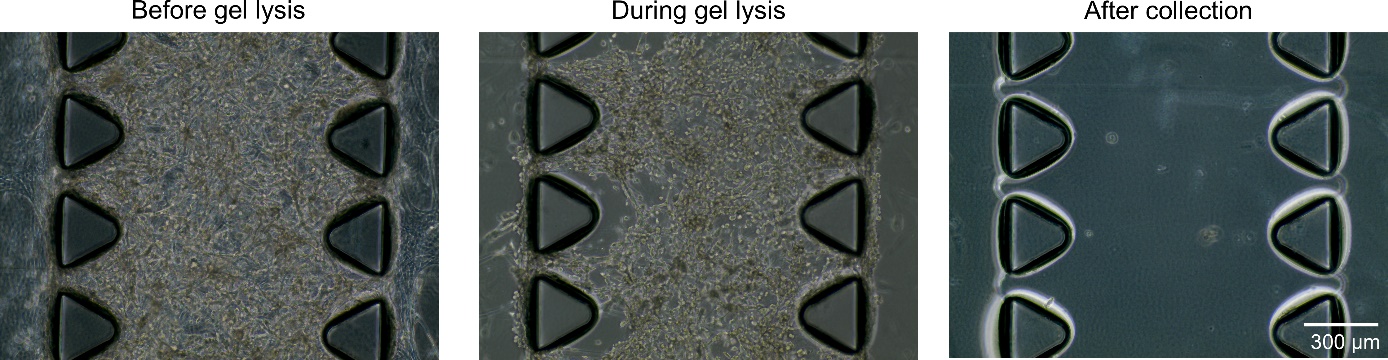


**Supplementary Figure 11. Representative brightfield images for vascularized heart-on-a-chip before gel lysis, during gel lysis using a collagenase solution (0.7 mg/mL), and after collection** (Scale bar: 300 μm).


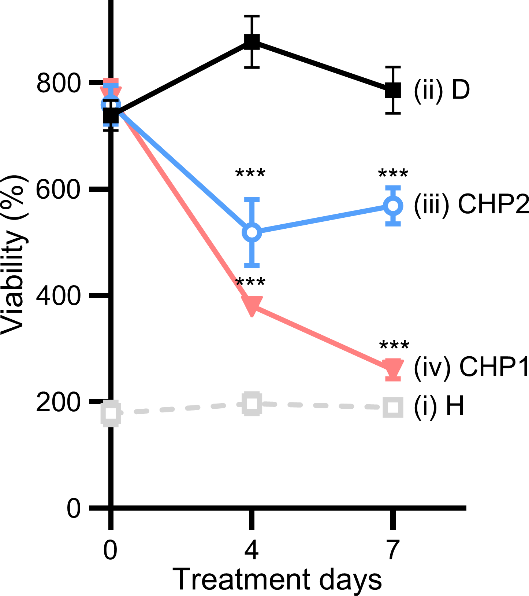


**Supplementary Figure 12.** **BNP secretion analysis in media collected from vascularized heart-on-a-chip tissues under various conditions from days 1 to 7**; healthy heart-on-a-chip (H, i), myocardial ischemia-on-a-chip without treatment of CHP-conjugated and Alamandine-loaded liposome (D, ii), with daily treatment of CHP-Lip/Alam at 250 µg/mL (CHP1, iii), and 500 µg /mL (CHP2, iv) (*n* = 4 chips). One-way ANOVA with post hoc Dunnett’s test was used to compare the difference between CHP1 or CHP2 and D; ***, *p* < 0.001.


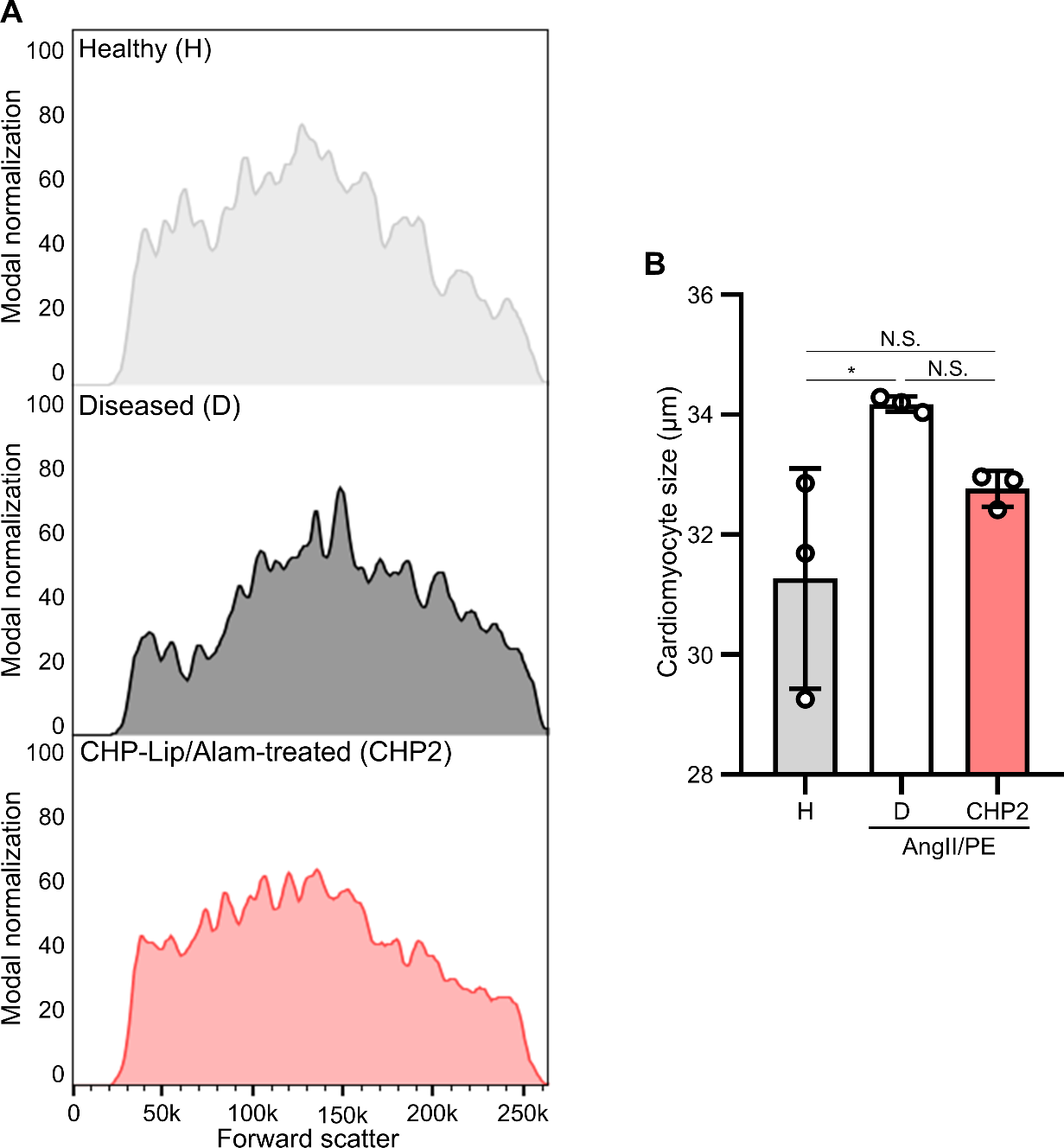


**Supplementary Figure 13. Cardiomyocyte (cTnT positive cells) size measurements extracted from vascularized heart-on-a-chip models under various conditions on day 7 using flow cytometry.** (A) Forward scatter (FSC-A) histogram of cardiomyocytes extracted from the vascularized heart-on-a-chip platform. (B) Quantification of cardiomyocyte size based on flow cytometric FSC-A values calibrated with size reference beads. One-way ANOVA with post hoc Tukey’s test was used to compare the differences between each condition after the normality test. Each point represents data from a single sample, which was prepared by pooling *n* = 3 chips from *n* = 3 independent experiments. N.S., not significant; *, *p* < 0.05.

**
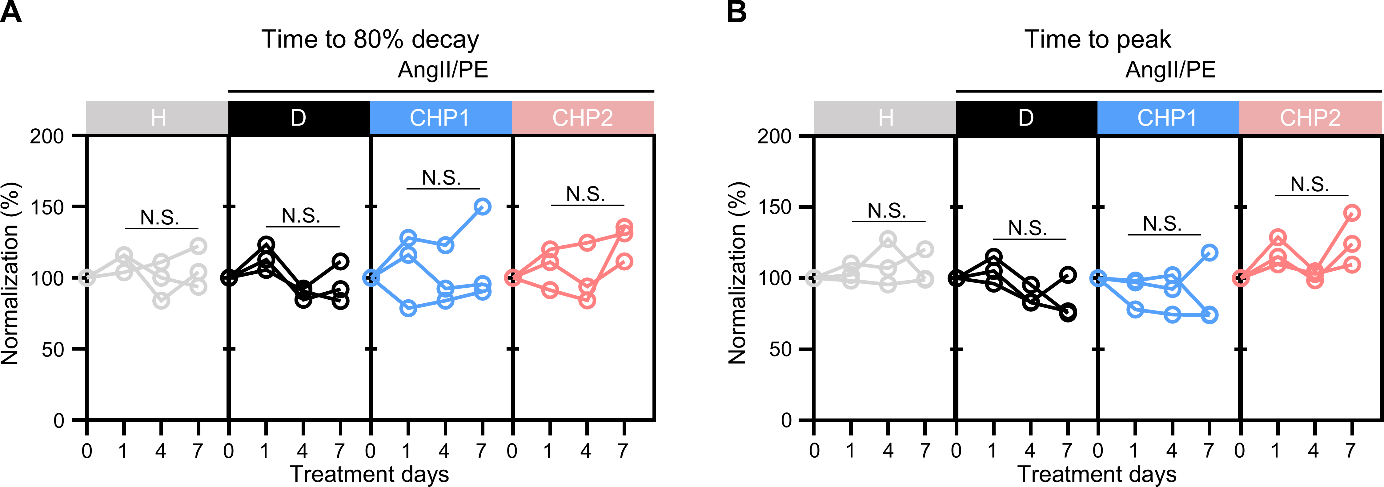
**

**Supplementary Figure 14. Profiles of Ca^2+^ transient parameters (80% decay time and time to peak) in healthy conditions without CHP-Lip/Alam treatment (“H” panel) and the myocardial ischemia conditions after treatment with different concentrations of CHP-Lip/Alam (“D, CHP1, and CHP2” panels in AngII/PE group) in the 3D heart-on-a-chip.** Each point represents data from each chip. Total *n* = 30 ROIs in 3 independent chips. All the data were normalized to the data from each chip before CHP-Lip/Alam treatment on day 0. Two-way ANOVA with post hoc Dunnett’s test (B, C, D, E) was used to compare each condition to its respective baseline (Day 0) after the normality test; N.S., not significant.


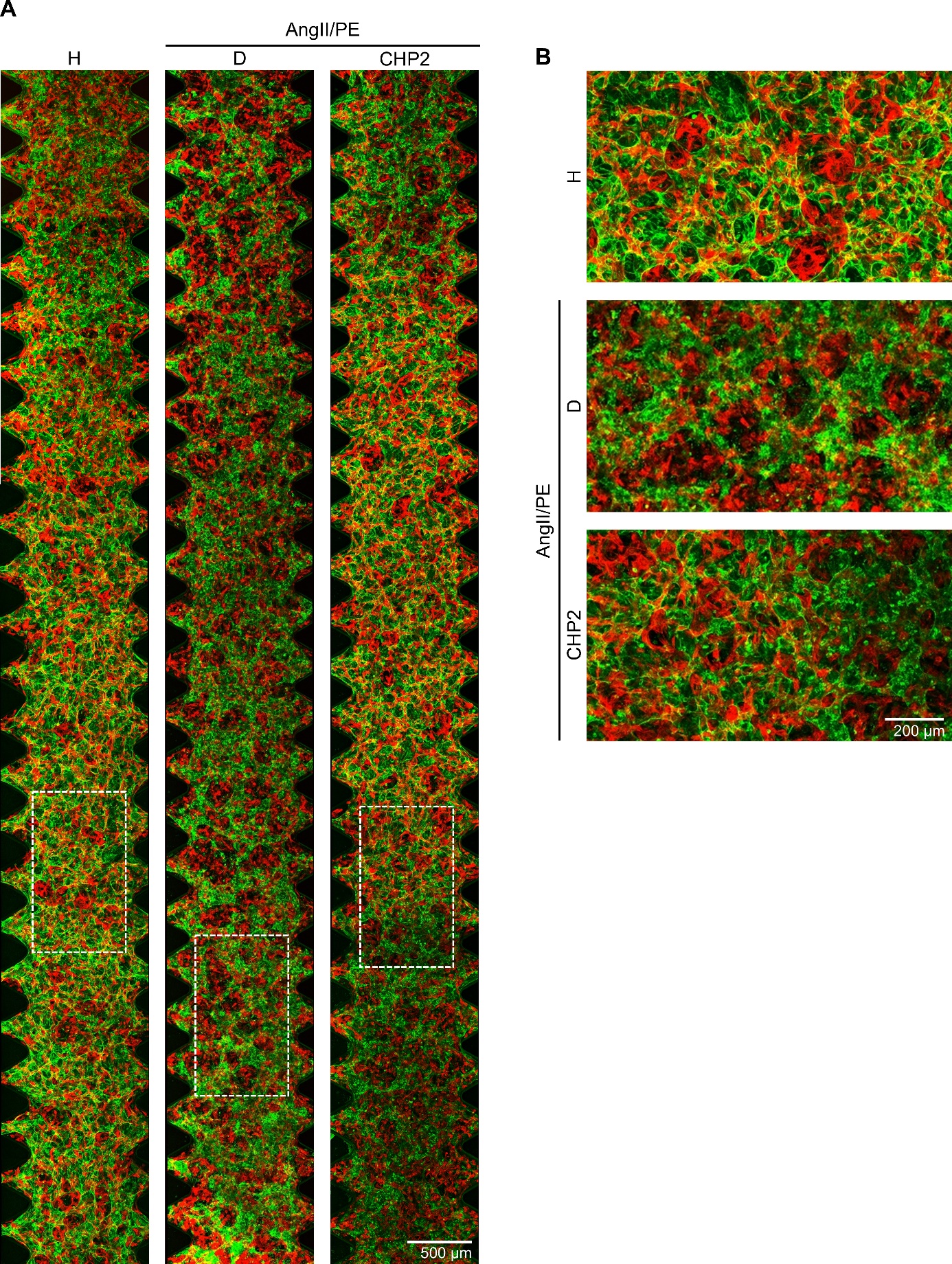


**Supplementary Figure 15. Whole-chip immunofluorescence imaging of engineered vascularized heart-on-a-chip at Day 14 (7 days post-treatment with CHP-Lip/Alam)**. (A) Tile-scanned maximum intensity projection image showing the entire heart-on-a-chip under three different conditions: H (healthy), D (diseased, myocardial ischemia without CHP-Lip/Alam treatment), and CHP2 (myocardial ischemia treated with 500 µg/mL of CHP-Lip/Alam). Red: cTnT-positive cardiomyocytes, green: CD31-positive vasculature, Scale bar: 500 µm. (B) Magnified view of the region highlighted by the white dashed box in (A). Scale bar: 200 µm.

**Supplementary Table 1. Gene name, gene symbol, and assay ID from TaqMan to assess gene expression by RT-qPCR.**

| **Gene name** | **Gene Symbol** | **Assay ID** |
| --- | --- | --- |
| Ryanodine receptor 2 | RYR2 | Hs00181461_m1 |
| ATPase sarcoplasmic/endoplasmic reticulum Ca^2+^ transporting 2 | ATP2A2 | Hs00544877_m1 |
| Transforming growth factor beta 1 | TGFB1, | Hs00998133_m1 |
| Actin, alpha 2, smooth muscle, aorta | ACTA2 | Hs00426835_g1 |
| Collagen type I alpha 1 | COL1A1 | Hs00164004_m1 |
| Vascular endothelial growth factor A | VEGFA | Hs00900055_m1 |
| Angiopoietin 1 | ANGPT1 | Hs00919202_m1 |

**Supplementary Video 1.** Spontaneous beating of engineered myocardium showing direct physical interaction with vessels in vascularized heart-on-a-chip (GFP: GCaMP Ca^2+^ indicators from CMs, RFP: RFP expressing HUVECs.

**Supplementary Video 2.** Introduction of fluorescently-labeled NPs into vessels of vascularized and perfusable heart-on-a-chip (GFP: GFP expressing endothelial cells, Red: fluorescently-labeled nanoparticles).

**Supplementary Video 3.** Spontaneous Ca^2+^ transients in GcaMP6f-expressing CMs within the healthy heart-on-a-chip model, recorded at baseline (day 0) and on days 1, 4, and 7 post-treatment.

**Supplementary Video 4**. Spontaneous Ca^2+^ transients in GcaMP6f-expressing CMs within the engineered heart-on-a-chip model under AngII/PE-induced ischemic conditions, recorded at baseline (day 0) and on days 1, 4, and 7 post-treatment.

**Supplementary Video 5**. Spontaneous Ca^2+^ transients in GcaMP6f-expressing CMs within the engineered ischemic heart-on-a-chip model following 7-day treatment with CHP-Lip/Alam (500 µg/mL), recorded at baseline (day 0) and on days 1, 4, and 7 post-treatment.

**References**

[1] P. W. Burridge, E. Matsa, P. Shukla, Z. C. Lin, J. M. Churko, A. D. Ebert, F. Lan, S. Diecke, B. Huber, N. M. Mordwinkin, J. R. Plews, O. J. Abilez, B. Cui, J. D. Gold, J. C. Wu, *Nature Methods* **2014**, 11, 855.

[2] Y. Zhang, A. Le Friec, Z. Zhang, C. A. Müller, T. Du, M. Dong, Y. Liu, M. Chen, *Materials Today* **2023**, 70, 237.

[3] C. Hajal, G. S. Offeddu, Y. Shin, S. Zhang, O. Morozova, D. Hickman, C. G. Knutson, R. D. Kamm, *Nature Protocols* **2022**, 17, 95.

[4] S. Zhang, Z. Wan, G. Pavlou, A. X. Zhong, L. Xu, R. D. Kamm, *Advanced Functional Materials* **2022**, 32, 2206767.
